# Marimo for monitoring and filtering of aquatic environments

**DOI:** 10.1007/s11356-025-37259-6

**Published:** 2025-12-19

**Authors:** Neil Phillips, Thomas C. Draper, Andrew P. Geary, Kathryn Lamb-Riddell, Darren M. Reynolds, Joshua A. C Steven, Freya Radford, Abdul R. Farooq, Andrew Adamatzky

**Affiliations:** 1 Unconventional Computing Laboratory, University of the West of England, Bristol, UK; 2 Institute of Bio-Sensing Technology, University of the West of England, Bristol, UK; 3 School of Architecture and Environment, University of the West of England, Bristol, UK; 4 Centre for Research in Biosciences, University of the West of England, Bristol, UK; 5 School of Applied Sciences, University of the West of England, Bristol, UK; 6 Centre for Machine Vision, University of the West of England, Bristol, UK

**Keywords:** Biomimicry, Bioengineering, Biological repository, Diatoms

## Abstract

*Aegagropila linnaei* (commonly known as “Marimo”) forms dense spheres of intertwined algae filaments capable of capturing and retaining a wide range of particles and microorganisms (up to 2 mm in length) from freshwater environments. This study explores the potential of Marimo as a sustainable tool for environmental monitoring and water filtration. We present a novel, non-destructive method for extracting entrapped materials (including microplastics, sediments, and indicator species) and evaluate various analytical techniques for characterising the extracted content. A prototype Marimo-based filtration system was developed, demonstrating significant reductions in microbial contaminants within a recirculating water system. These findings highlight the advantages of using Marimo as a low-cost, eco-friendly solution for long-term aquatic ecosystem monitoring and remediation.

**Supplementary Information:**

The online version contains supplementary material available at 10.1007/s11356-025-37259-6.

## Introduction

The increasing prevalence of microplastic pollution, nutrient loading, and microbial contamination in freshwater systems presents a growing challenge for environmental sustainability and public health. Conventional water monitoring and treatment technologies, while effective, often require significant infrastructure and maintenance, prompting the search for low-cost, nature-based alternatives.

Recent research has increasingly focused on sustainable water treatment technologies, including the exploration of agricultural and industrial waste materials as eco-friendly filtration media. Badawi et al. (2024b, a) demonstrated the effectiveness of modified rice husk waste-based filters for wastewater treatment, showing promising reuse potential in pilot-scale applications. Similarly, Hassan (2024) employed date palm fiber filters for greywater treatment, offering a biodegradable and low-cost alternative. Choksi and Amarsheebhai (2022) explored the use of waste casting sand as an adsorbent in the secondary treatment of produced water, highlighting its industrial applicability.

In the realm of microplastic mitigation, Alwan et al. (2024) reviewed state-of-the-art strategies, emphasising identification techniques and emerging technologies, and identified gaps in scalable removal methods, advocating for integrated approaches combining filtration, adsorption, and biodegradation.

Overall, there is a strong trend toward sustainable, low-cost, and multifunctional materials derived from waste. However, challenges remain in scalability, long-term performance, and integration into existing infrastructure. While microplastic removal technologies are progressing, further development is needed for widespread implementation.

Marimo (*Aegagropila linnaei*), a naturally occurring spherical aggregation of filamentous algae, offers a unique structural advantage due to its dense radial filament network and slow growth rate, which together facilitate long-term entrapment of microplastics, sediments, and microorganisms without requiring artificial substrates. This study investigates the potential of Marimo as a sustainable tool for aquatic environmental monitoring and filtration, building on recent advances in eco-engineering and green remediation technologies (Patel et al. (2025); Ahmed et al. (2025); Tan et al. (2024)).

Marimo show promising potential in lake restoration through adaptive nitrate removal. Their granular structure enhances sedimentation, while photosynthetic activity supports nutrient uptake. Marimo autonomously adjusts denitrification pathways in response to environmental nitrate levels, initially boosting nitrate reduction and later shifting to assimilatory nitrate reduction as nitrate concentrations decline. This dynamic response improves nitrogen removal efficiency and supports stable microbial communities (Wang et al. (2024)). Marimo’s low energy requirements, cold tolerance, and adaptability make it a sustainable, nature-based solution for mitigating eutrophication and improving lake water quality.

**Fig. 1:**
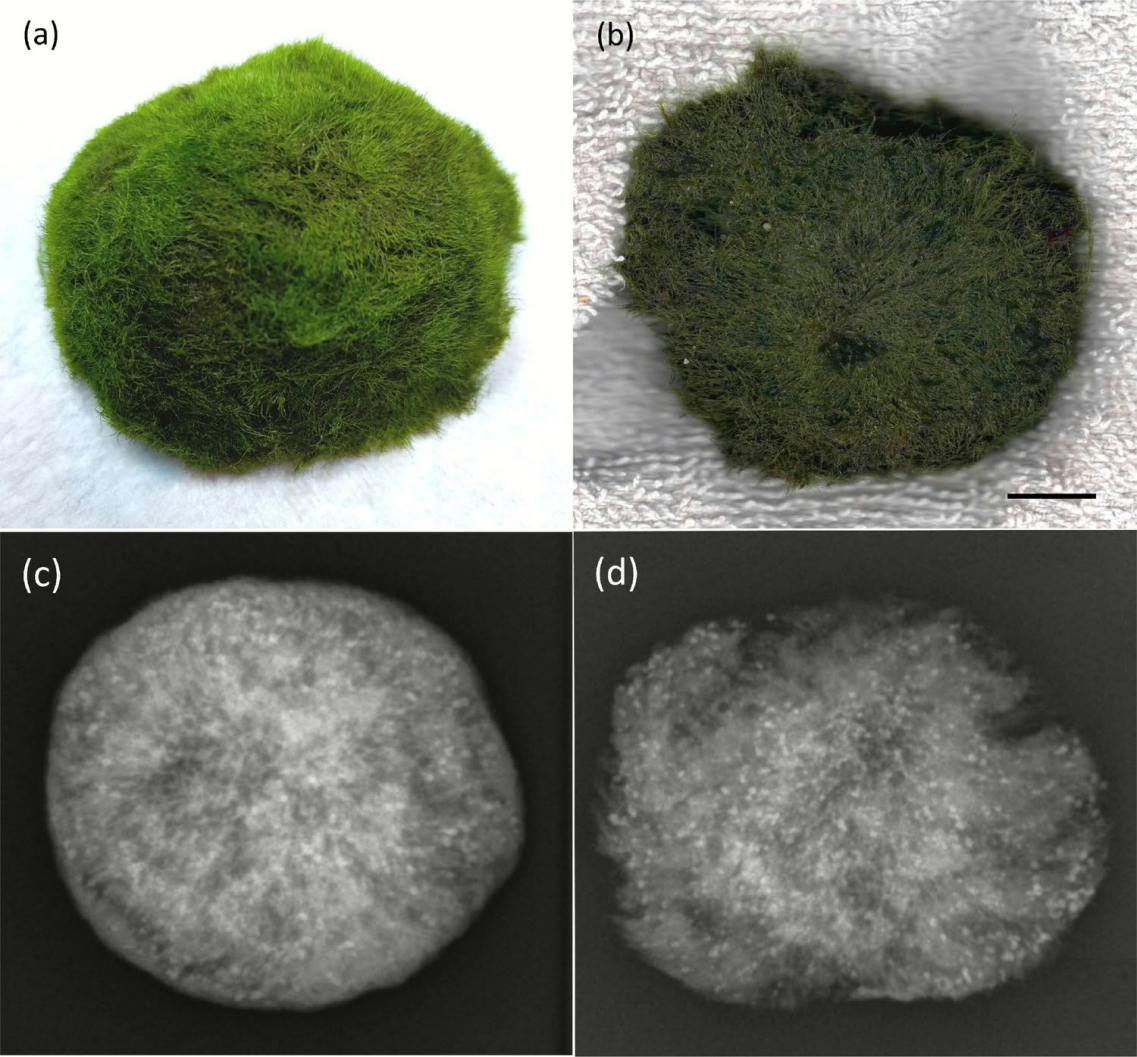
Marimo balls of ~ 50 mm diameter. **(a)** Photograph of plan view; (**b)** flat-bed scan of cross-sectioned body; (**c)** X-ray of whole body; (**d)** X-ray of cross-sectioned body (DR 600, Agfa HealthCare Ltd, BE). Scale bar: 10 mm

Experimental testing demonstrates that algae filaments in *Aegagropila linnaei* (commonly known as “Marimo”) are promising elements for environmental monitoring systems. *A. linnaei* forms spherical structures (Cooper (2018); Bryant and Irvine (2016); Umekawa et al. (2021)), as shown in (Fig. 1a), through natural rolling and self-adhesion of filamentous algae in turbulent freshwater currents (Togashi et al. (2015); Boedeker (2010); Ogata et al. (2021); Hayashi et al. (2018); Sano et al. (2016)). Images of both the exterior and the cross-section of a Marimo are presented in (Fig. 1). The cross-sectional image (Fig. 1b) reveals that the filamentous structure is continuous throughout the sphere, although Marimo of larger diameters may contain an internal hollow compartment (Nakayama et al. (2021)). Marimo have also been observed to rise and sink in their natural habitats due to the generation of gas bubbles that adhere to their filaments (Phillips et al. (2019); Cano-Ramirez et al. (2018); Phillips et al. (2022)).

Marimo was selected for this study due to its unique structural and ecological characteristics, which make it particularly well-suited for passive environmental sampling. Its dense filamentous architecture, formed through natural rolling, creates a robust three-dimensional matrix capable of entrapping a wide range of particulate matter. Marimo also exhibits an exceptionally slow growth rate of only a few mm per year (Soejima et al. (2009)), contributing to the long-term stability of its structure and enhancing its ability to retain microplastics, sediments, and microorganisms over extended periods. Unlike other filamentous algae, its unusual radial filament arrangement (Soejima et al. (2009)) facilitates internal deposition of contaminants without the need for artificial scaffolding or substrates, making it a promising candidate for sustainable, low-maintenance monitoring and filtration applications.

In this study, we present prototype applications of Marimo for environmental monitoring and filtration. These applications aim to stimulate further research and discussion, with the goal of developing sustainable, cost-effective bioengineering systems.

## Materials and methods

Marimo specimens were sourced from Live Aquarium Plants Ltd, UK. Based on the observation of filaments arranged in a “radial type” pattern (Nakai et al. (2021); Horiguchi et al. (1998); Einarsson (2014)) in (Fig. 1b) and discussions with the supplier, it is believed that the specimens originated from Lake Svityaz in Ukraine (Boedeker et al. (2010)).

The Marimo were kept in glass tanks (Boyu model EC-600; 66 L capacity; dimensions, 600 mm L × 300 mm W × 500 mm H) filled with municipal water supplemented with 0.1 mL/L (v/v) of a commercially available fertiliser (TNC Complete, The Nutrient Company, UK), providing phosphorus and nitrogen. When not in use, the Marimo were maintained under a day/night cycle while avoiding direct sunlight.

A pump was used to recirculate the water/fertiliser mixture at a flow rate of 500 L/h. The mixture was refreshed every 4 weeks. All experiments were conducted at room temperature (18 to 22 °C).

### Extracting environmental samples from Marimo

The filamentous nature of the alga that Marimo are composed of enables it to collect and accumulate debris from their local aquatic environment (including fine sediment from the surface of lake beds and suspended particulates) (Nakayama et al. (2021); Nakai et al. (2021)). Rinsing Marimo in clean, free-flowing water can be used to partially extract trapped debris. To improve extraction efficiency, centrifugal force was applied by centrifugal rotation of the Marimo while submerged in water using a magnetic stirrer. Several parameters were optimised:

- Enclosure diameter: glass laboratory beakers with internal diameters ranging from 75 to 150 mm
- Water height: beakers filled with water to a height between 70 and 160 mm
- Stir bar length: PTFE-coated magnetic stirrer bars ranging from 6 to 100 mm
- Stir bar ends: PTFE-coated stir bars with 2 to 8 ends

**Fig. 2:**
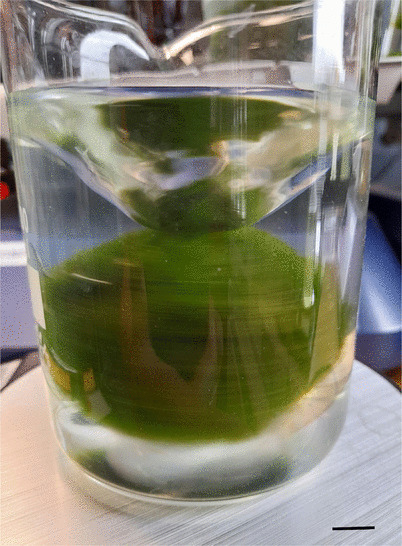
Material was extracted from Marimo (~ 50 mm diameter) by subjecting them to centrifugal rotation at a speed of (204 ± 40) rev min^−1^ along the primary axis and (43 ± 33) rev min^−1^ along the secondary (tumbling) axis, using a stirrer bar rotating at 760 rev min^−1^. Scale bar: 10 mm

The optimal configuration for a Marimo (~ 50 mm diameter) was a glass laboratory beaker of 400 mL capacity, 75 mm internal diameter, 100 mm height, filled with 300 mL of water and a magnetic stir bar with two ends of 35 mm length and 9 mm diameter (see Fig. 2). The bar was rotated by a magnetic stirrer (N-MX-3K, Anzeser Ltd, China) at the maximum achievable speed while maintaining stable rotation for ~ 1 h.

After rotation, the Marimo was carefully removed from the beaker without disturbing the settled debris at the bottom.

A video of the white stirrer bar (magnet encapsulated in PTFE) half painted black rotating while submerged in water at 240 frames per second (FPS) with a high-speed digital camera (Google Pixel 4a, Google LLC, USA) was recorded. The video was viewed at 30 FPS (8× slow down) to reveal the speed of rotation. A Marimo was air-dried for approximately 48 h, and then one hemisphere was sprayed four times (allowing each coat to dry before applying the next) with a black graphite spray (Graphit 33, Kontakt-Chemie, Germany). The “Janus-Marimo” was then recorded at 240 FPS while rotating to reveal rotation speed along the primary axis (aligned with the base of the beaker) and the secondary axis (tumbling motion).

### Optical inspection of Marimo balls

An unprocessed (not subjected to centrifugal rotation) Marimo (~50 mm diameter) and a second Marimo of similar size (from the same supplier and batch), which had been subjected to centrifugal rotation for 1 h, were placed side by side. Both were illuminated with the same ultraviolet lamp (500 lx LED spot, wavelength 395 nm) positioned equidistantly between them at a height of 100 mm.

The Marimo were photographed simultaneously (A20, Samsung, South Korea). The hue and saturation of the resulting digital image were adjusted to enhance the contrast between surface artefacts and the algal filaments. Image analysis was performed using the Counting Measurement feature in Photoshop 2022 (version 23.3.2, Adobe Systems Inc., USA), following the identification of individual artefacts based on their high colour contrast against the background (Chen et al. (2018); Zhang et al. (2014); Putman et al. (2005); Slusarewicz et al. (2016)).

### Gravity sedimentation and organic dissolution of extracted material

After rotation, both the Marimo and the stirrer bar were carefully removed from the glass beaker. The remaining liquid mixture was left undisturbed for an additional 24 h to allow suspended particles—entrained by the turbulence of the rotating water to either settle or float. Floating organic material (less dense than water) was gently removed from the surface using a micropipette, air-dried for 48 h, and examined.

The bulk of the remaining liquid was then decanted, and the residual fluid was allowed to stand for an additional 24 h. Excess liquid above the settled material was again removed with a micropipette. The remaining residue was air-dried for a further 48 h and weighed.

This residue was found to contain both organic matter and the desired inorganic matter. To isolate the inorganic fraction, the organic matter was removed using a method previously reported in the literature that preserves the size, shape, and spectral characteristics of polymers (Nuelle et al. (2013); Hanvey et al. (2017)). Specifically, the residue was mixed with 30% H_2_O_2_ in glassware at room temperature (18 to 22 °C) until frothing subsided. The solution was then gradually heated to ~90 °C while periodically stirring. Above approximately 70 °C H_2_O_2_ is rapidly consumed; additional H_2_O_2_ must be added to continue the digestion of organic matter, as described by Mikutta et al. (2005).

The resulting solution was transferred to a 50-mL centrifuge tube (with conical base) and left to stand for about 1 h to allow the inorganic material to settle. Excess fluid was removed with a micropipette, and the remaining material was gently dried in an electric oven at 80 °C for 24 h before being weighed.

The dried samples were examined under an optical microscope (SMZ-2B, Nikon, Japan) at 50× magnification. Images of selected particles were captured using a 5MP MU500-HS digital microscope camera (AmScope Ltd, US), and scale bars were added using AmScope Version x64 software.

As an alternative to using hydrogen peroxide, sodium hypochlorite (NaClO at 7.5%) heated to 60 to 70 °C has also been reported (Pfeiffer and Fischer (2020)) as an effective agent for digesting both soft and hard organic tissues.

### Particle size distribution of inorganic residue

The particle size distribution of the inorganic residue obtained from Marimo was measured using laser diffraction (Mastersizer 2000 (10.1108/prt.1998.12927fad.017), Malvern Instruments Ltd, UK) across a range of 0.02 to 2000 µm. In laser diffraction measurements, the particles move through a beam of focused laser light. The particles scatter the light at an angle, the value of which is inversely proportional to particle size (Ryżak and Bieganowski (2011)).

To prepare the samples for analysis (following the removal of organic material and drying, as described in “Gravity sedimentation and organic dissolution of extracted material”), a slurry was created by adding a few drops of 5% sodium hexametaphosphate solution. Three replicates of each sample were measured, and the average values are reported.

In total, particle size distributions were measured for five residue samples in a liquid dispersion of 400 mL deionised water (contained in a 600-mL tall-form glass beaker, 80 mm diameter, Simax Ltd, Czech Republic, to minimise the required sample mass). Three samples (M1-A, M1-B, M1-C) were collected by rotating Marimo for 1 h, and one sample (M100-A) for 100 h. The fifth sample (“Whole”) was prepared by gradually dissolving the entire Marimo in 30% hydrogen peroxide at ~80 °C until the addition of hydrogen peroxide no longer produced bubbling.

### Microplastic analysis

Three methods were used to analyse microplastics in samples extracted from Marimo after the removal of organic matter using hydrogen peroxide, as described in “Gravity sedimentation and organic dissolution of extracted material”.

#### Analysis by optical spectroscopy

Microscope images of microplastic and nanoplastic fibres, beads, and particles extracted from samples M1-D, M1-E, M1-F, M1-G, M1-H, M1-I, and M1-J were taken using three cameras (Pixel 4a 16MP Google USA; A20 13MP Samsung JP; and MU500-HS 5MP AmScope, USA). Illumination at two wavelengths (395 nm and 365 nm) was used to optimise contrast between fibres and the background.

#### Analysis of microfibres by Raman spectroscopy

**Fig. 3:**
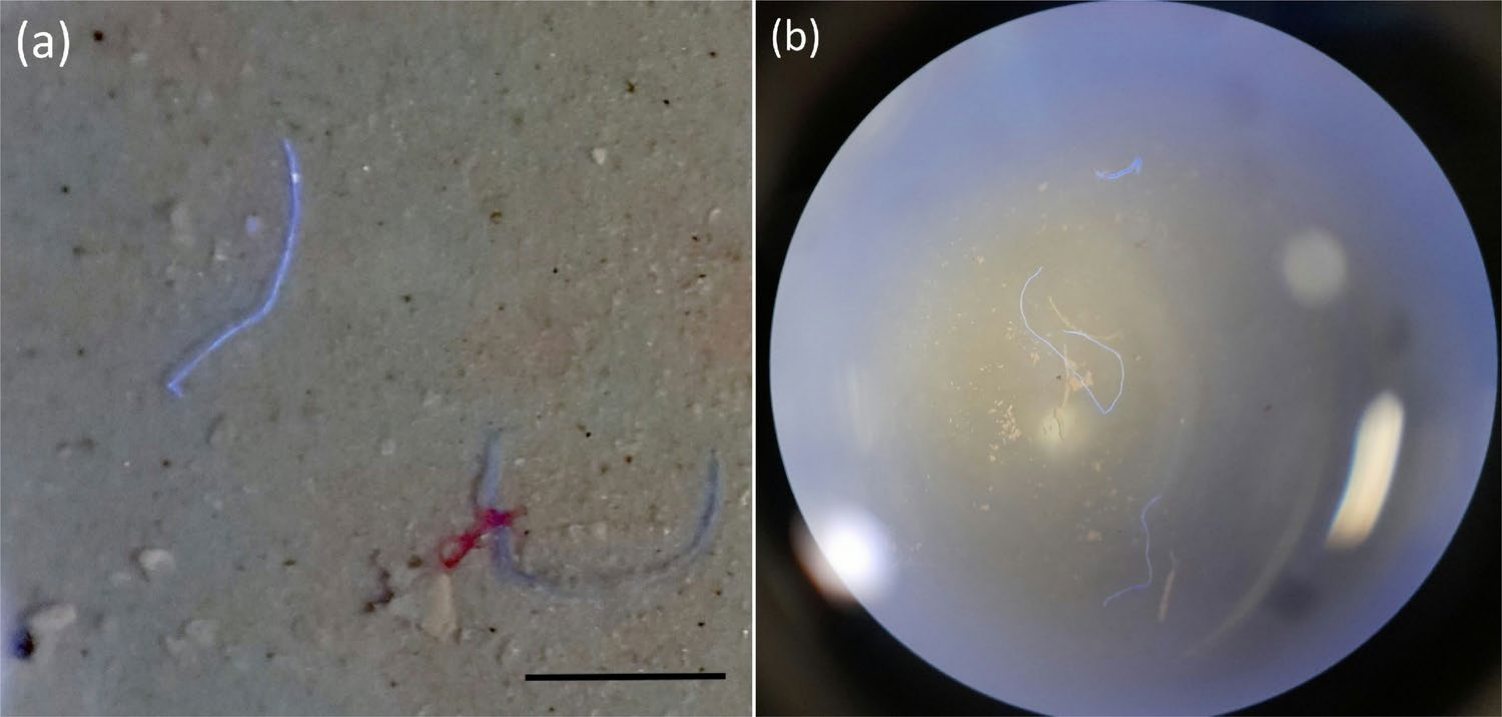
Manual fibre separation (**a**) fibres mixed with other residue on a glass Petri dish. Scale bar 500 µm (**b**) separated fibres (and minimal particles of residue) in a glass bottle (1 mL)

Dried samples were carefully examined under an optical microscope (SMZ-2B, Nikon, Japan) at 50× magnification. A combination of ultraviolet (365 nm) and “cool white” light (450 to 700 nm) was used to visualise both fluorescent and non-fluorescent fibres (see Fig. 3a). Seven exemplar fibres were manually isolated using ultra-fine tweezers (model 07287-07, Cole-Parmer, UK). The separated fibres were temporarily stored in a glass bottle (1 mL) (see Fig. 3b), before being analysed with Raman spectroscopy (Gillibert et al. (2019); (Institute 2022)).

The chemical composition of individual microfibres was determined by their vibrational Raman spectra, which were recorded using a Horiba LabRAM HR Evolution Confocal Raman Microscope, equipped with a 1200 line/mm grating, using an Olympus M Plan x50, NA 0.75 objective lens. Raman spectra were recorded in the range 100 cm^−1^ for laser excitation of ~50 mW @ 785 nm. Total integration time was typically 100 s. Lab6 software was used to process spectra from 1 to 3500 cm⁻¹, removing any fluorescence background and reducing noise. Identification was performed by comparing spectra with the Wiley Science Solution’s KnowItAll Raman database (https://sciencesolutions.wiley.com/knowitall-analytical-edition-software/). Spatial resolution was ~3 µm, and spectral resolution was 3 cm.

#### Analysis by Fourier transform infrared (FTIR) spectroscopy

Micro- and nanoplastics vary significantly in size (0.001 to 5000 µm) in the Food Chain (CONTAM) (2016).

Manipulating objects smaller than 200 µm with mechanical tweezers is challenging (Alogla et al. (2015)). Therefore, dense medium separation (DMS) (Li et al. (2018)) was used to separate plastic particles from other debris based on density differences.

A zinc chloride solution with a density of 1.1 g/cm^3^ was prepared by dissolving 2 mL zinc chloride (ZnCl_2_, 1.8 g/cm^3^) in 9 mL deionised water (H_2_O, 15 MΩcm, 1.0 g/cmg/cm^3^).

**Fig. 4:**
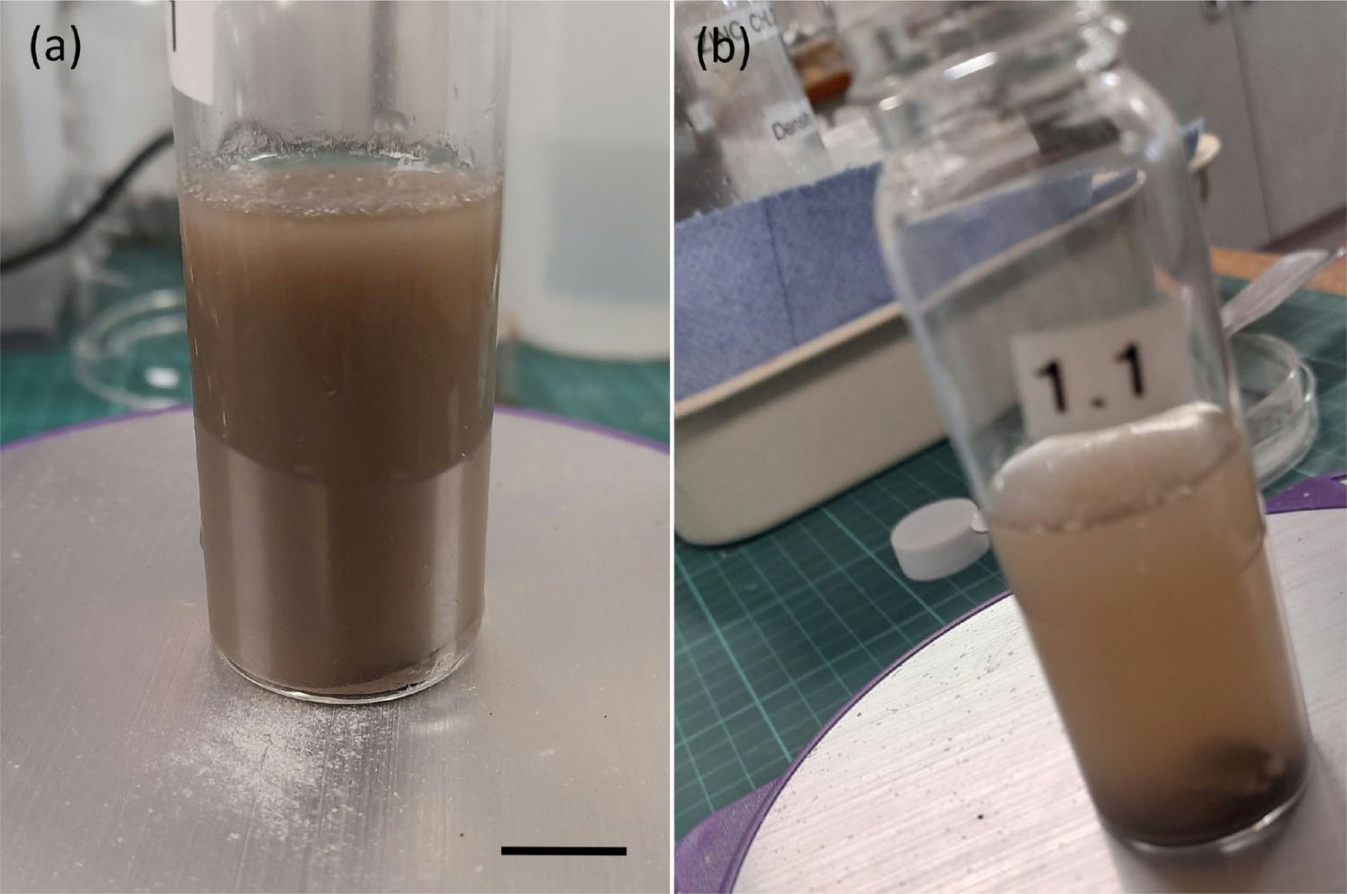
Microplastic separation: (**a)** residue mixed with zinc chloride–water solution (scale bar: 10 mm); (**b)** after settling

The seven samples (M1-D, M1-E, M1-F, M1-G, M1-H, M1-I, M1-J) were combined (minus manually removed fibres) and thoroughly mixed with the zinc chloride-water solution in closed glass bottle (20 mL) with magnetic stirrer (bar of 6 mm length, 3 mm diameter, polytetrafluoroethylene coating) for 5 min (see Fig. 4a). The mixture was then left to stand for 15 min to provide particles time to sink, float, or remain in suspension (see Fig. 4b). A micropipette was thoroughly washed with deionised water and used to transfer ~1 mL from the top of the mixture into a glass watch glass (50 mm diameter, which was covered with another watch glass), and the liquid was allowed to evaporate. The dry residue was examined using Raman spectroscopy (Gillibert et al. (2019)).

The remaining liquid was carefully poured into a glass laboratory vacuum filtration apparatus (300 mL funnel with 250 mL reservoir, Yamai, China) connected to a pump (VP2, KNF, Germany) fitted with a 0.4-µm pore size nuclepore polycarbonate hydrophilic track-etched filter membrane (47 mm diameter, product code: 111107, Whatman Plc, UK). Analysis of the dry residue on the filter with Raman spectroscopy was unable to provide a reliable identification of particles due to the noisy spectrum.

The dry residue was examined with Fourier transform infrared (FTIR) spectroscopy, following the methodologies described by Cabernard et al. (2018), Chen et al. (2020), and de Jesus Piñon-Colin et al. (2018). For each sample, the dry residue was resuspended in a 20-mL glass vial containing a 50% ethanol solution. Subsamples were taken and deposited onto silver filters (3 µm pore size, Sterlitech, Washington, USA) through an 8-mm diameter silicon ring for FTIR analysis. Sub-sample quantities were measured by weighing the vials before, and the subsamples were taken. Filters were analysed using FTIR (Spotlight 200i, Perkin Elmer). Five-point samples were taken across each filter on targeted particles identified as potential microplastics (Hartmann et al. (2019)). Each particle was scanned in triplicate at a spectral resolution of 4 cm⁻¹ over the range 4000–600 cm⁻¹ and analyzed for spectral matches using Spectrum 10 software. (PerkinElmer) (Radford et al. (2021)).

A spectral map with an area of 2 × 2 mm was also scanned at a random position on the filter for each sample. The filter was scanned in reflectance mode with 3 scans per pixel at a pixel resolution of 100 µm and spectral resolution of 4/cm in the range of 4000 to 600/cm. Spectral maps were processed and analysed using siMPle software (Aalborg University and Alfred Wegener Institute (2022)). The Aalborg University pipeline using raw and first derivatives was used, with a minimum particle size of one pixel (100 µm). Particles were identified using the siMPle automated IR database (version 1.0.1) and classified by size and mass (as an estimate based on particle volume, polymer density, and an assumed ellipsoid 3-dimensional shape).

### Micro-organism analysis

Samples of material extracted from Marimo were processed to remove most organic matter using hydrogen peroxide, as described in “Gravity sedimentation and organic dissolution of extracted material”. The remaining content was carefully examined under an optical microscope (BH200P, Brunel Microscopes Ltd, UK) at 400× magnification (objective 40×, eyepiece 10×). Images were recorded using a 5.1 MP digital microscope camera (MU500-HS, AmScope Ltd, USA).

### Environmental sustainability

To assess the potential impact of high-speed rotation (subjected to centrifugal extraction) on Marimo, the daily volume of gas generated by individual specimens was measured for 4 days prior to and 6 days following a 1-h period of centrifugal extraction.

**Fig. 5:**
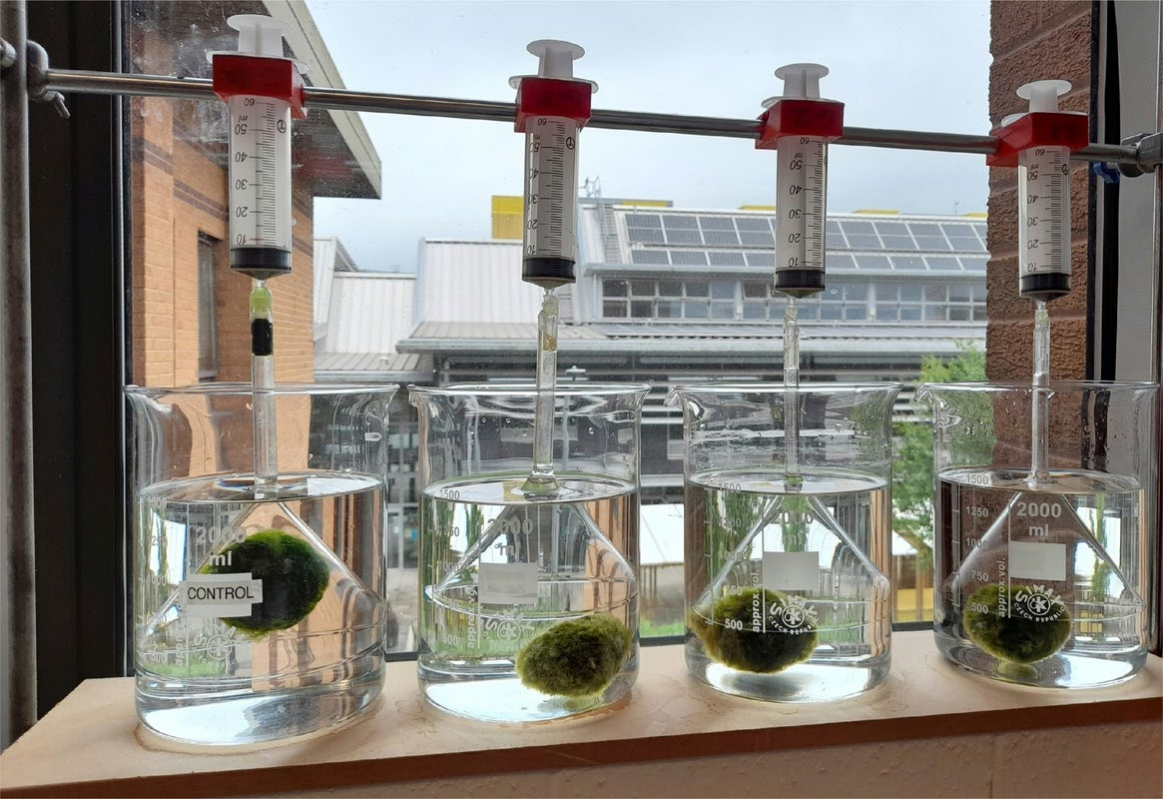
Photograph of the quad rate of gas generation measurement rig

Each Marimo was placed in a transparent, water-filled 2-L glass beaker, beneath a glass funnel (120 mm diameter) connected to a 60-mL syringe. This setup allowed any gas bubbles released by the Marimo to rise into the funnel and be captured (see Fig. 5). The change in gas volume inside the syringes was recorded while maintaining a constant water level.

Four funnels were operated simultaneously: three experimental and one control. All beakers were filled with tap water containing 0.1 mL/L of the previously mentioned commercial fertiliser (providing phosphates and nitrates). After 4 days of baseline measurements, the three experimental Marimo were subjected to 1 h of high-speed rotation, while the control was left unaltered. Gas evolution measurements continued for six additional days.

At the end of the experiment, all Marimo were kept in darkness for 24 h to serve as a further control; no gas was collected during this period. The experimental setup was placed on a raised windowsill and exposed to indirect sunlight. The measurement accuracy for gas volume was ± 0.5 cm^3^.

### Material collection and loss rate

**Fig. 6:**
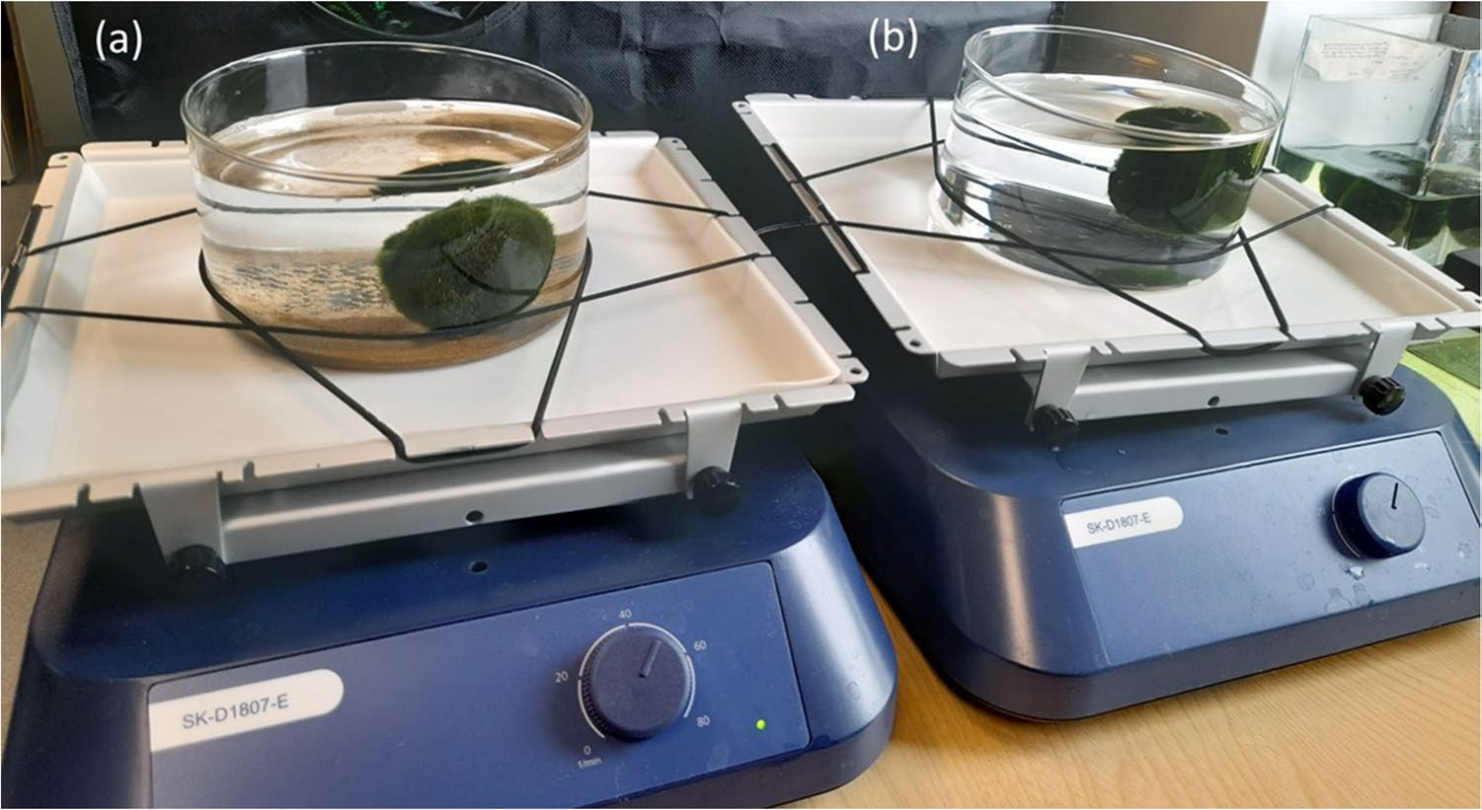
Photograph of Marimo on 3D orbital shakers: (**a)** dish contains fine sand, (**b)** empty dish

Pyrex borosilicate glass crystallising dishes without a spout (140 mm diameter, 75 mm height, 900 mL capacity, product code 11766592, DWK Life Sciences Ltd, Germany) were centrally placed on a pair of 3D orbital shakers (model SK-D1807-E, Camlab Choice Ltd, UK). The dishes were secured using non-slip mats and bungee cords (see Fig. 6).

Approximately 25 g of fine sand (collected from the dunes of Sand Bay, Somerset, UK) was weighed and added to one dish with 750 mL of deionised water (15 MΩcm). The second dish, serving as a control, was filled with 750 mL of deionised water only.

Two Marimo (~50 mm diameter, M1-N, M1-O) previously subjected to 1 h of high-speed rotation were placed in the respective dishes. Both dishes were swirled at 50 rev/min on 3D orbital shaker for ~20 h.

After swirling, the Marimo were carefully removed, and the water was allowed to stand for 24 h. Any floating organic material (less dense than water) was carefully removed from the surface with a micropipette. The bulk of the remaining water was carefully decanted from the dishes and discarded. The dishes were then left for an additional 24 h, after which excess water above the settled material was removed with a micropipette. The remaining residue was air-dried for 48 h and weighed.

The proportions of inorganic and organic matter were determined using the method described previously. The particle size distributions of the collected and extracted inorganic matter and the original beach sand were measured using a Mastersizer 2000 (Malvern Instruments Ltd, UK).

### Marimo filtration

#### Experimental setup

**Fig. 7:**
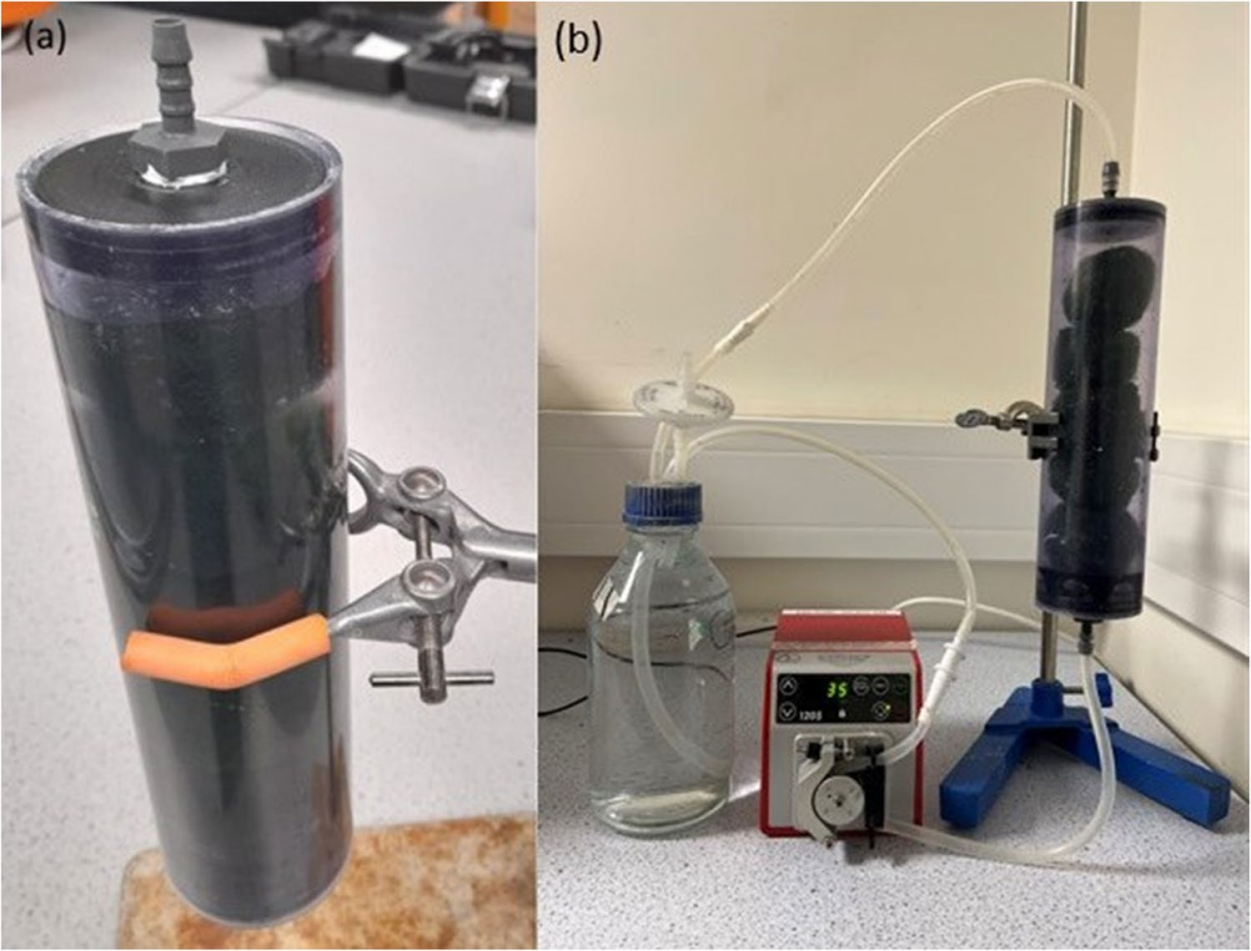
Photographs of filtration measurement: (**a)** filter column, (**b)** complete test rig

Five Marimo (~50 mm diameter) were contained in a laboratory test filter column consisting of a 700-mL PVC column (63 mm internal diameter, 230 mm length, (see Fig. 7a)). The column was connected to a double-ported flask via a multi-channel peristaltic pump (model 505S, Watson-Marlow Fluid Technology Solutions Ltd, UK).

Water was collected from environmentally derived surface water held in an urban drainage pond, N 51° 29.56′ W 2° 32.39′. The levels of the indicator species in the test water notably increased during the summer months (May–October; Table S5 in the Electronic Supplementary Information (ESI)). Mean monthly concentrations of enterococci ranged from 185 to 1223 CFU per 100 mL during this period. In contrast, viable enterococci counts were markedly lower between November and April, ranging from 8 to 70 CFU per 100 mL. The bacterial loading used to challenge the Marimo filter in this study was sampled during the summer months.

One litre was added and circulated through the Marimo filter for a period of 24 h at a flow rate of 35 mL/min (see Fig. 7b).

Standard biological and physiochemical water quality indicators, including *E. coli*, enterococci, heterotrophic plate counts (HPCs), ammonium, nitrite, and nitrate, were measured in the test water before and after 24-h circulation. A 100-mL aliquot of water was collected and analyzed using the membrane filtration technique. Filters were placed onto selective and differential agar for enumeration; enterococci were quantified using Slanetz and Bartley agar. 1 (OxoidTM, Basingstoke, UK); for presumptive *E. coli*, Membrane Lactose Glucuronide Agar (OxoidTM, Basingstoke, UK).

A control filter column, identical in design but without Marimo, was used to account for any pathogen reduction attributable to the experimental apparatus itself.

All microbiological and physicochemical measurements were performed in triplicate.

#### Ion chromatography

Ion chromatography (850 Professional IC Anion, Metrohm Ltd, Switzerland) was used to quantify the levels of anions and cations relevant to the nutrients of interest with regard to water quality and microbial growth: phosphate (PO_4_^3−^), nitrite (NO_2_^−^), nitrate (NO_3_^−^), and ammonium (NH_4_^+^).

Water samples were collected from the reservoir and immediately filtered through a 0.2-µm filter to prevent any further microbial activity. Aqueous samples were then loaded onto an autosampler in open-top tubes and auto-injected.

For anion analysis, a sodium carbonate (3.2 mM/L) and sodium bicarbonate (1.0 mM/L) mobile phase was used throughout. Background conductivity of the mobile phase was suppressed by a cation exchanger and regenerated using a dilute sulphuric acid (150 mM/L) and oxalic acid (100 mM/L) solution.

For cations, a nitric acid (0.7 mM/L) and dipicolonic acid (1.7 mM/L) mobile phase was used.

IC-certified standards (Fisher Scientific, UK) were used to generate calibration curves, from which the concentrations of ions in the water samples were interpolated.

#### Total carbon analysis

Total carbon analysis (enviro TOC, Elementar Ltd, Germany) was used to quantify the levels of total inorganic carbon (TIC) and total organic carbon (TOC) in the water samples.

Samples were collected from the environmentally derived surface water and immediately filtered through a 0.45-µm membrane to prevent further microbial activity. The filtered aqueous samples were then loaded into open-top tubes and auto-injected into the analyser.

#### Dissolved oxygen

Dissolved oxygen (DO) levels in the environmental water circulated through the Marimo filter system were measured using an optical DO metre (HQ10, Hach Company, USA). The metre was calibrated prior to use with oxygen-saturated air to ensure accurate readings.

#### Radio-frequency identification

**Fig. 8:**
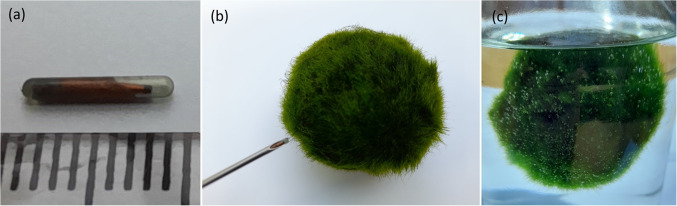
Photographs of (**a)** an RFID microchip encapsulated in a bioglass capsule, (**b)** inserting the capsule into a Marimo with an applicator, (**c)** Marimo floating with an RFID tag in the centre

A radio-frequency identification (RFID) tag, in the form of a microchip encapsulated in a bioglass capsule (1.25 mm × 7 mm) (see Fig. 8a), was manually inserted into the centre of a Marimo with an applicator (see Fig. 8b). The Marimo was monitored following insertion.

## Results

### Extracting environmental samples from Marimo balls

Several factors were found to influence the stability of rotating Marimo, including the diameter of the container, the height of the water, and the size and shape of both the stirrer bar and the Marimo itself. Notably, when the container diameter was only slightly larger than the Marimo, it rotated more stably in a minimal orbit, allowing for higher rotational speeds.

The presence of the Marimo significantly altered the flow dynamics of the water. Instead of the typical vertical “funnel” vortex observed at the water surface vortex (Halász et al. (2007)), a horizontal vortex formed, with increasing depth toward the centre, as illustrated in Fig. 2. If the stirrer bar was too long or had multiple ends, it often collided with the Marimo, disrupting the rotation.

It was observed that the total mass of material removed from the Marimo increased with the duration of rotation. A Marimo (~50 mm diameter) rotated at a speed of (204 ± 40) rev/min on the primary axis and (43 ± 33) rev/min on the secondary axis (tumble), with the stirrer bar rotating at 760 rev/min.

No visible harm was observed in ten Marimo specimens over a 6-month monitoring period after the removal of material (1 h of high-speed rotation). On the contrary, Marimo that had undergone cleaning floated slightly higher in the water and appeared visually healthier, likely due to the removal of debris and dead biomass.

### Optical inspection of Marimo balls

**Fig. 9:**
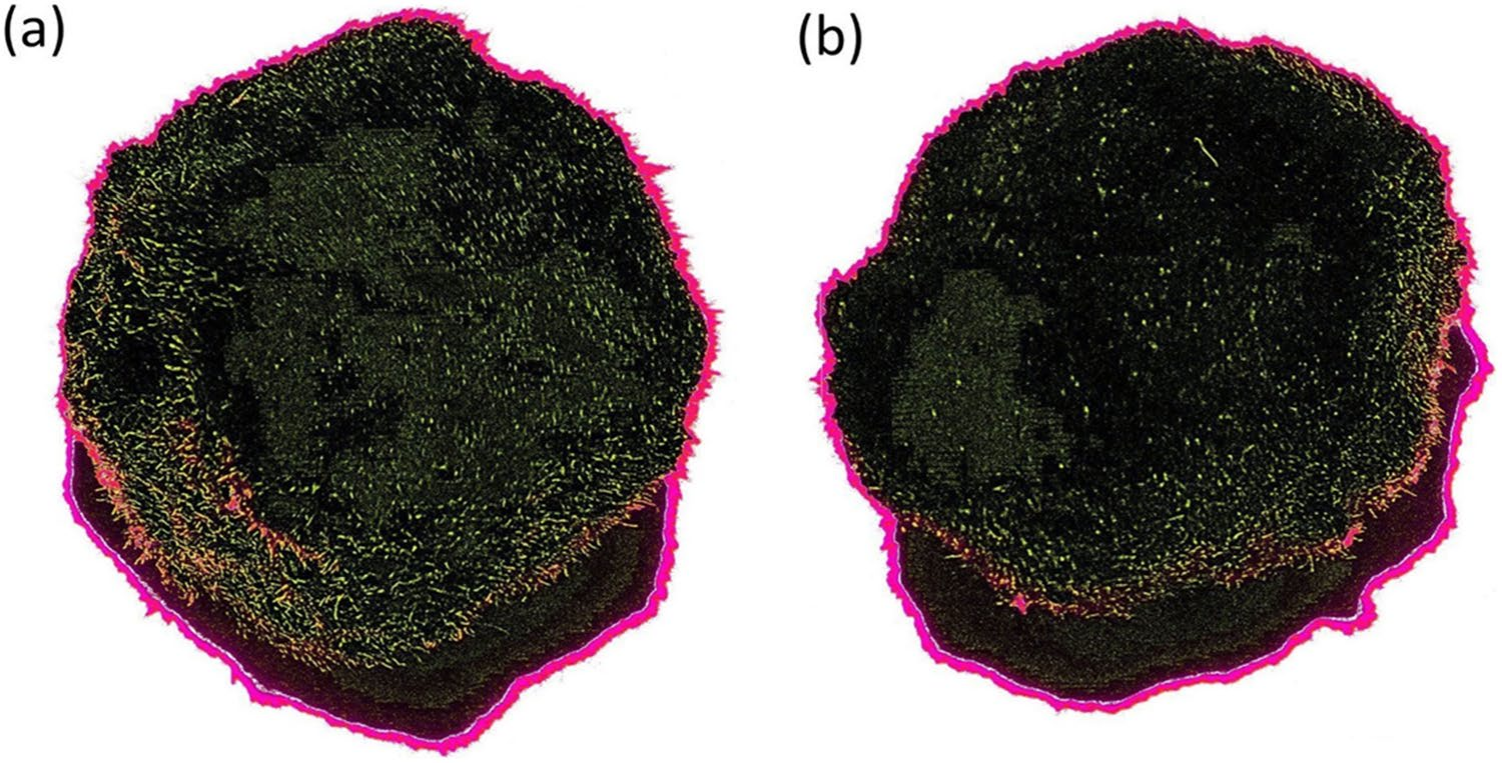
Digitally enhanced images of two Marimo: (**a)** untreated, (**b)** after being subjected to centrifugal rotation for ~ 1 h

Figure 9 shows a digitally enhanced image of two Marimo that were simultaneously and equally illuminated with the same ultraviolet lamp. Image analysis software calculated that the unprocessed Marimo (Fig. 9a) had 2917 artefacts, whereas the Marimo subjected to centrifugal rotation (Fig. 9b) had 1550 artefacts. Furthermore, artefacts on unprocessed Marimo were observed to be generally larger in physical size than on Marimo subjected to centrifugal rotation.

### Analysis of extracted material

**Fig. 10:**
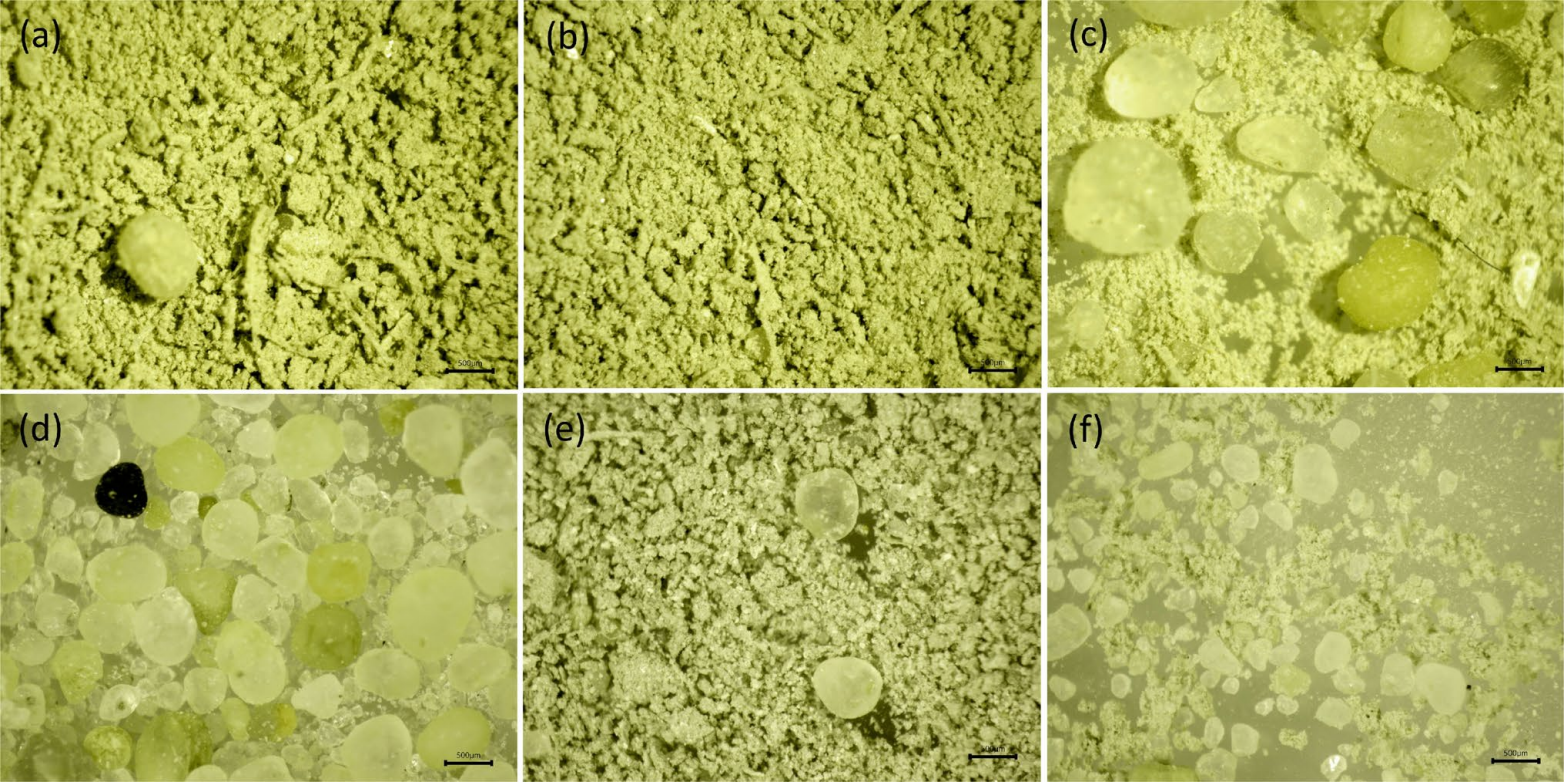
Exemplar microscope images of inorganic material illuminated at 365 nm, 5 MP MU500-HS camera: (**a)** sample M1-A, (**b)** sample M1-B, (**c)** sample M1-C, (**d)** sample “Whole”, (**e)** sample M1-J, (**f)** sample M1-F

Mass of (inorganic and organic) material removed from Marimo by centrifugal force and filtration separation is shown in Table 1. Exemplar microscope images of inorganic material are shown in (Fig. 10).

**Table 1:** Material removed from Marimo (~ 50 mm diameter, 1-h centrifugal rotation). Statistical analysis on the amount of inorganic vs organic mass revealed no statistical difference across the group (paired *t*-test, *p* = 0.672). Correlation analysis suggests that organic and inorganic matter collection was driven by a common factor (Pearson’s *r* = 0.82, *p* = 0.002)

Marimo reference	Total debris (g)	Inorganic mass (g)	Organic mass (g)	Inorganic:organic
*M1-A*	0.265	0.150	0.115	1.304
*M1-B*	0.550	0.332	0.218	1.523
*M1-C*	0.280	0.152	0.128	1.188
*M1-D*	0.530	0.360	0.170	2.118
*M1-E*	0.180	0.130	0.050	2.600
*M1-F*	0.420	0.160	0.260	0.615
*M1-G*	1.090	0.500	0.590	0.847
*M1-H*	0.305	0.100	0.205	0.488
*M1-I*	0.220	0.065	0.155	0.419
*M1-J*	0.530	0.309	0.221	1.398
Average	0.437	0.226	0.211	1.250
Standard deviation	0.253	0.133	0.139	0.672

### Particle size distribution of inorganic residue

**Fig. 11:**
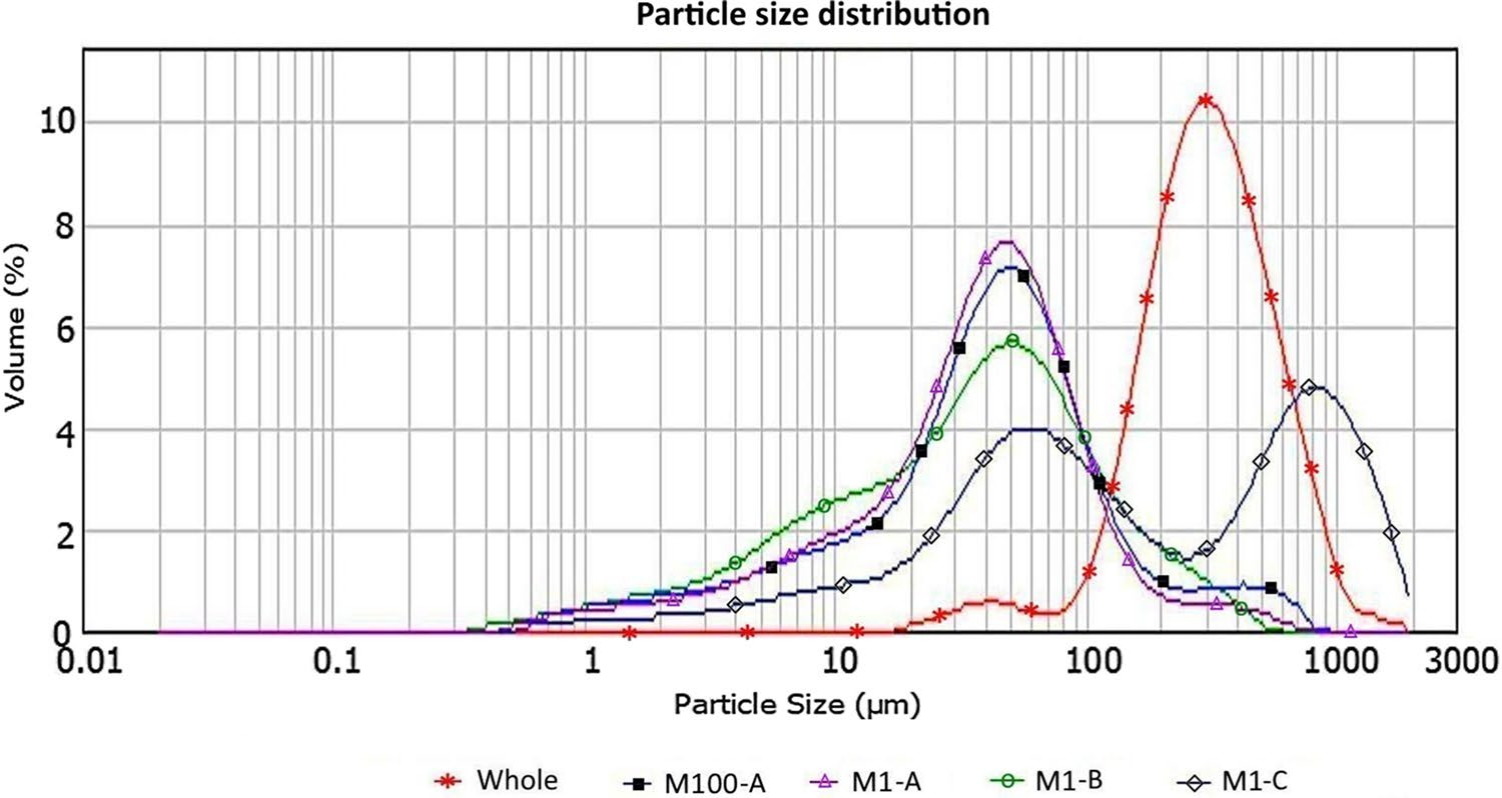
Particle size distribution of inorganic material obtained from Marimo and analysed with the Mastersizer 2000. Samples M1-A, M1-B, and M1-C after 1-h rotation, sample M100-A after 100 h rotation, and sample “Whole” (see ESI S1 for additional details)

Figure 11 shows the particle size distribution of residues obtained from Marimo subjected to centrifugal rotation (see ESI S1 for additional details). It can be seen that all of the samples that were spun for either 1 h or 100 h show a similar peak particle size of approximately 50 µm, with the exception of one which is bimodal and has an additional peak at 800 µm. This suggests that 1 h is a sufficiently long period of rotation to remove the vast majority of artefacts from a Marimo.

Figure 11 also shows the distribution of the particles recovered from a Marimo (labelled as “Whole”), which was not rotated but instead fully dissolved using the protocol reported in “Gravity sedimentation and organic dissolution of extracted material”. It can be seen that this also presents a bimodal distribution, with one peak at approximately 50 µm and another larger peak at 300 µm. The presence of this second peak suggests that the centrifugal rotation used to remove the particles from Marimo (see “Extracting environmental samples from Marimo”) is very effective for smaller particles but may leave larger particles trapped inside the Marimo.

### Analysis of extracted microplastics

Analysis of the microplastics extracted from Marimo was conducted using three spectroscopy techniques: optical, Raman, and FTIR.

#### Analysis by optical spectroscopy

**Fig. 12:**
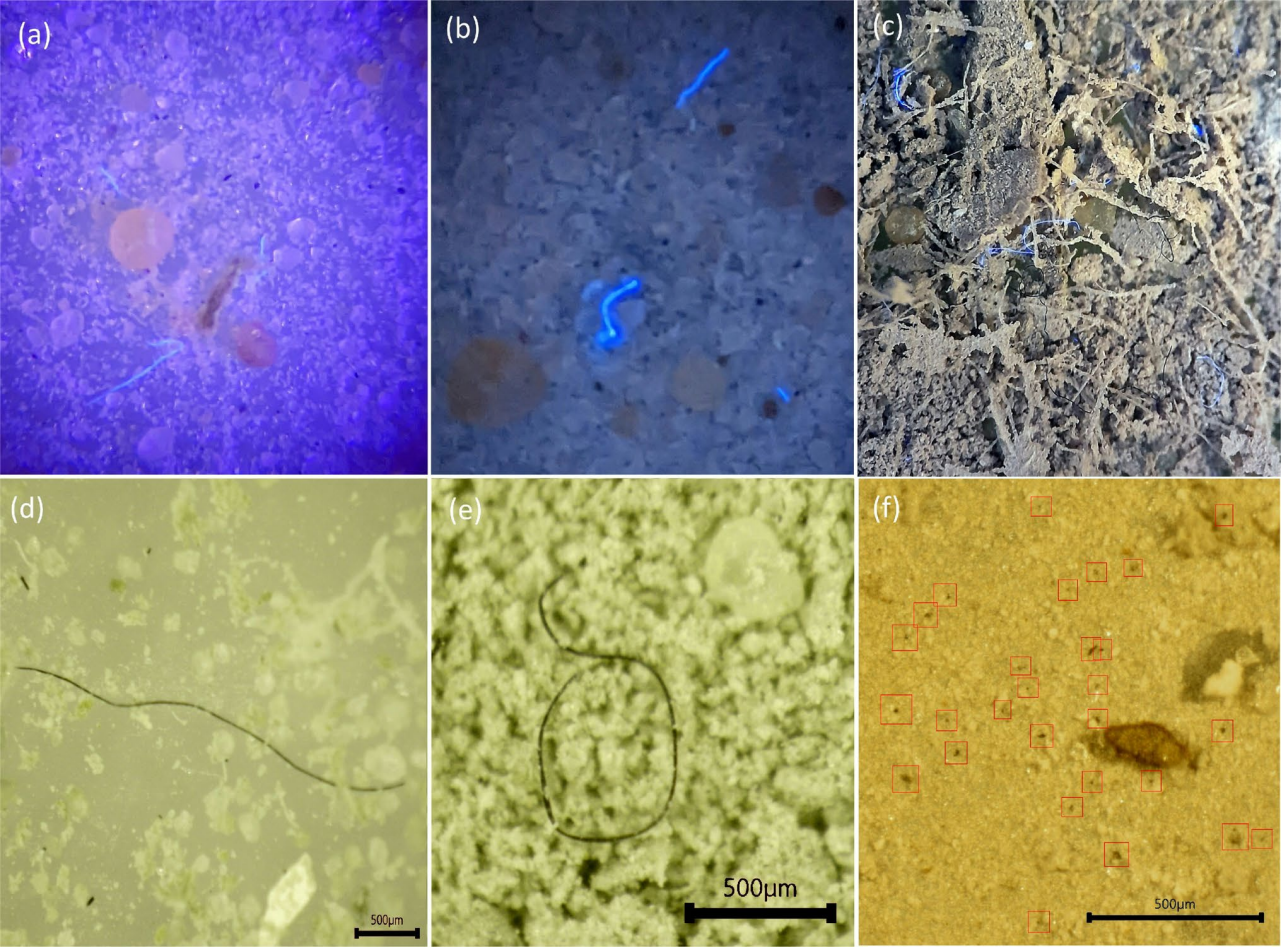
Microscope images of particles and fibres extracted from Marimo: (**a)** × 50 magnification, illumination at 395 nm with A20 Samsung; (**b)** × 50 magnification, illumination at 365 nm with Pixel 4a Google; (**c)** × 50 magnification, illumination at 365 nm with A20 Samsung; (**d)** sample M1-I illumination at 365 nm with MU500-HS AmScope; (**e)** sample M1-A illumination at 365 nm with MU500-HS AmScope; (**f)** possible microplastic beads in red squares, illumination “cold white” (450 to 700 nm) light with MU500-HS AmScope

Exemplar microscope images of micro- and nanoplastic fibres, beads, and particles extracted from samples M1-D, M1-E, M1-F, M1-G, M1-H, M1-I, and M1-J are shown in (Fig. 12). Three cameras (Pixel 4a 16MP Google USA, A20 13MP Samsung JP, and MU500-HS 5MP AmScope USA) and illumination at two wavelengths (395 nm and 365 nm) were tried to obtain the best contrast between fibres and background. A scale bar was added with the AmScope software.

#### Analysis by Raman spectroscopy

**Fig. 13:**
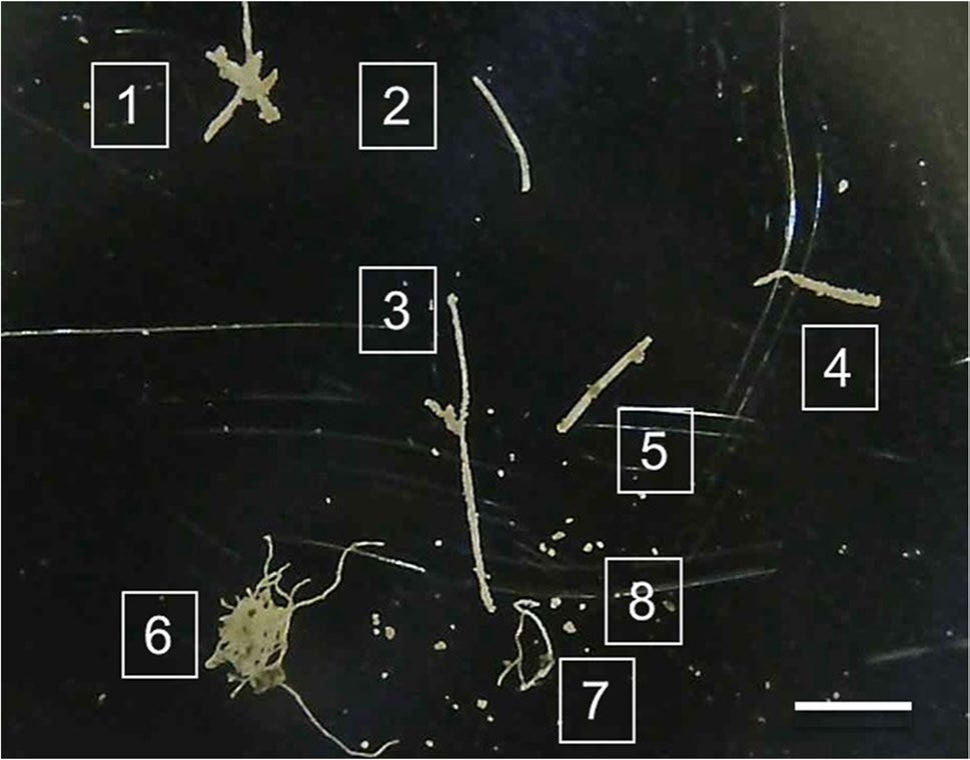
Microscope image of the fibres and particles analysed with Raman spectroscopy: (1) rough, irregular fibre cluster; (2) thin, smooth fibre; (3) thin, rough fibre; (4) irregular, rough fibre; (5) thick, smooth fibre; (6) thin fibre cluster; (7) thin, irregular fibre; (8) irregular, shiny particles. Scale bar: 1 mm

**Fig. 14:**
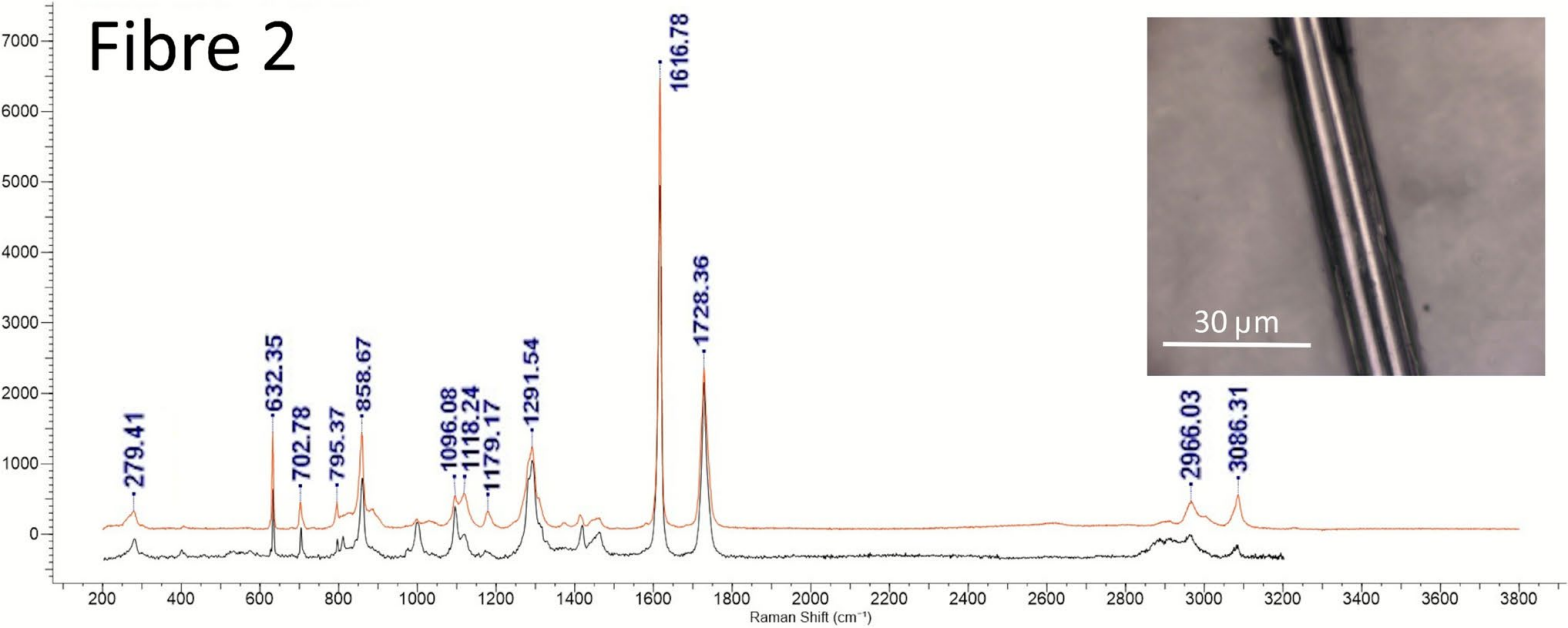
Fibre 2 Raman spectrum: Raman spectrum (black) and database spectrum (red). Raman spectrum: Raman peaks at 279.41, 631.66, 702.78, 858.00, 997.81, 1093.84, 1289.63,1417.21, 1614.45, 1726.28 cm^−1^. KnowItAll database ID match Polyethylene terephthalate (PET) 85.03%. Thin, smooth fibre. Probably PET

Optical microscope image of the fibres and particles on a clean aluminium metal substrate, shown in Fig. 13. The sample contained several single fibres and fibre clusters, and also a number of small particles. All fibres (1–7) were analysed by Raman spectroscopy. A selection of the particles (8) was also analysed. Representative spectra and image for Fibre 2 can be seen in Fig. 14, while details for Fibres 1, 3, 4, 5, 6, 7, and Particle 8 can be seen in Figures S3 to S9, in the ESI. The Raman results have additionally been summarised in Table S3, in the ESI.

#### Analysis by Fourier transform infrared (FTIR) spectroscopy

**Fig. 15:**
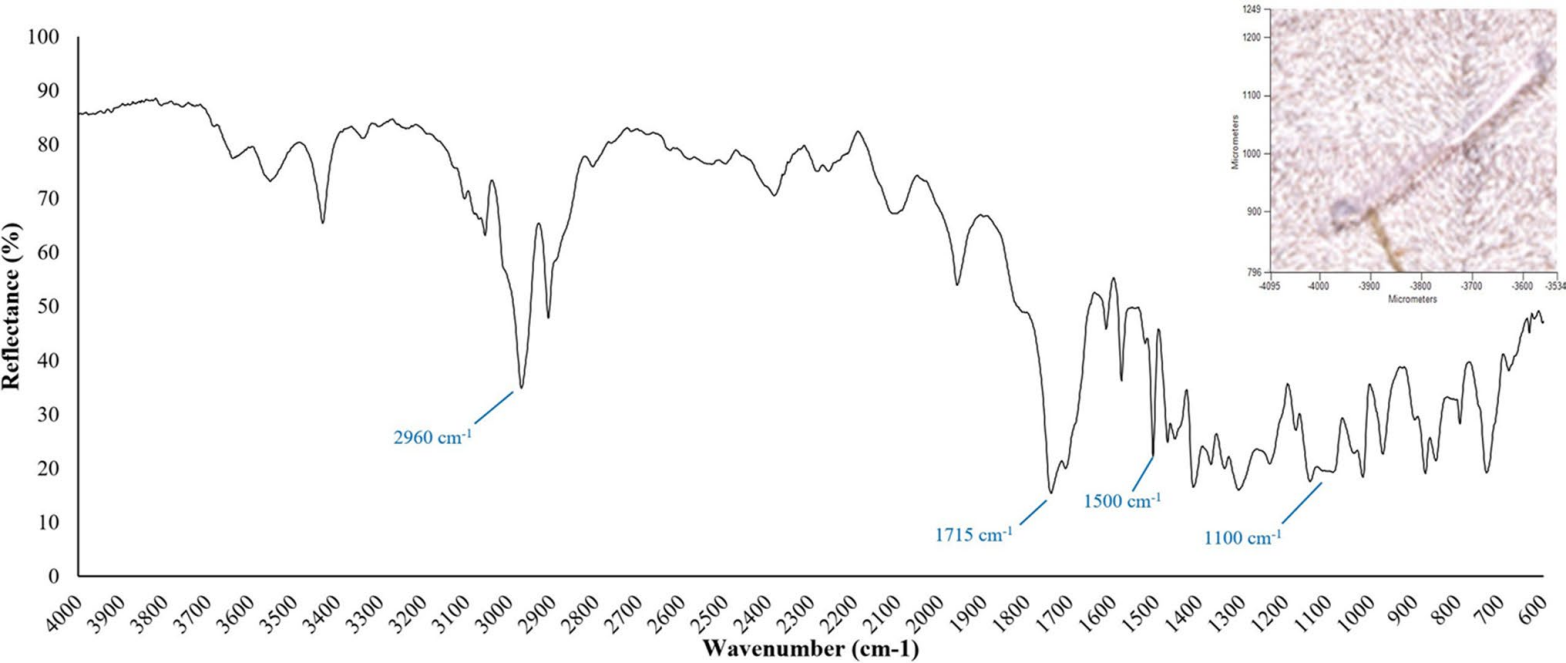
FTIR reflectance graph and image of a clear polyester fibre from Marimo sample M1-V (HQI = 0.96)

Five spot measurements were analysed from three samples of material extracted from Marimo; the findings are summarised in Table S4 in the ESI. An exemplar particle from sample M1-V (also listed in Table S4) and associated reflectance graph are shown in Fig. 15. The FTIR reflectance graphs of M1-W and M1-X (listed in Table S4) are shown in Figures S10 and S11, respectively, in the ESI.

**Fig. 16:**
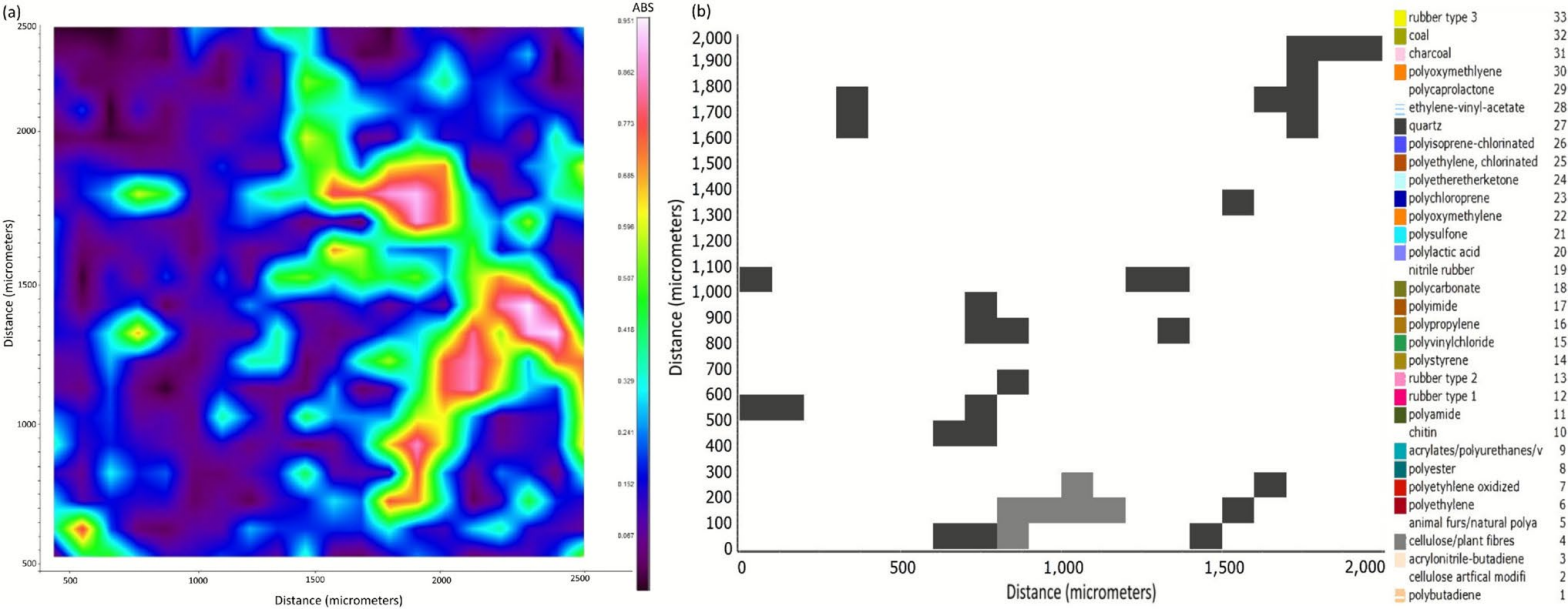
Scan of a 2 × 2 mm area of sample M1-X using Spotlight 200i FTIR: (**a)** infrared absorption map, (**b)** map of identified particles (quartz and cellulose)

Exemplars of infrared absorption maps from Spotlight 200i FTIR and identified particles from the siMPle software of a 2 × 2 mm area of sample M1-X are shown in (Fig. 16).

### Micro-organism analysis

**Fig. 17:**
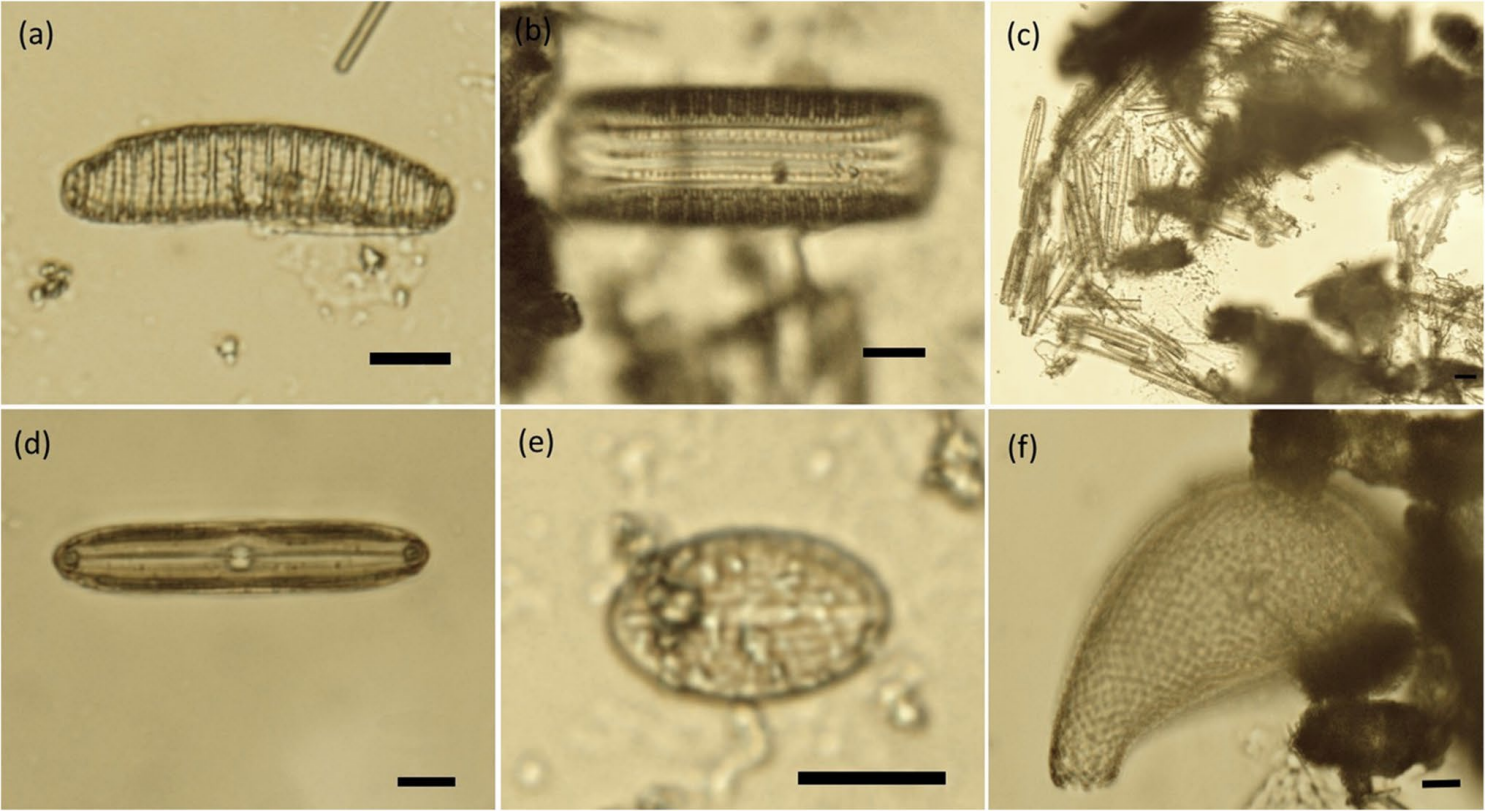
Microscope images (× 400) of microorganisms extracted from Marimo: (**a)** pennate species of diatom, (**b)** pennate species of diatom in girdle view, (**c)** diatom cluster, (**d)** pennate species of diatom, (**e)** *Cocconeis* genus of diatom, (**f)** testate amoebae, *Cyphoderia ampulla*. Scale bar: 10 µm

Six exemplar microscope images of microorganisms found in material extracted from Marimo are shown in (Fig. 17). Silica frustules of pennate species of diatom are shown in (Fig. 17a) (*Epithemia* sp.), (Fig. 17b) (*Epithemia* sp.) (girdle view), (Fig. 17c) (aggregation of pennate diatom frustules), (Fig. 17d) (*Pinnularia* sp.), and (Fig. 17e) (*Cocconeis* sp.). The testate amoebae, *Cyphoderia ampulla*, are shown in (Fig. 17f). The total number of individual organisms observed in the material extracted from Marimo was found to be variable in the order of 100 per Marimo subjected to centrifugal rotation for 1 h.

### Environmental sustainability

No change in the visible appearance of the Marimo (~50 mm diameter) was observed after being subjected to centrifugal rotation for 1 h. It was noted that they appeared to float slightly higher in the water after material was removed. Table 2 compares the daily volume of gas generated by Marimo before and after being subjected to centrifugal rotation. The measurements suggest that Marimo are not harmed by a single short period (1 h) of being subjected to centrifugal rotation. In fact, gas production actually increased 20%.

**Table 2:** Volume of gas generated daily by Marimo subjected to centrifugal rotation for 1 h, applied between days D4 and D5 (except control). There is a + 20.5% increase in gas production post-rotation. Analysis indicates a statistically significant increase in daily gas production after rotation (*p* = 0.044). The effect size is large (Cohen’s *d* = 1.26), suggesting a meaningful biological change. The control remains unchanged. *Average daily value over a 3-day period (weekend). Averages and standard deviations do not include control

**Day**	**Control (cm**^3^**)**	**M1-K (cm**^3^**)**	**M1-L (cm**^3^**)**	**M1-M (cm**^3^**)**	**Average (cm**^3^**)**	**Standard deviation**
D1	6.0	5.0	4.0	3.0	4.00	0.82
D2	5.0	4.0	3.0	2.0	3.00	0.82
D3	6.0	5.0	4.0	4.0	4.33	0.47
D4	6.0	5.0	5.0	3.0	4.33	0.94
D5*	6.0	6.0	4.3	3.7	4.67	0.97
D6*	6.0	6.0	4.3	3.7	4.67	0.97
D7*	6.0	6.0	4.3	3.7	4.67	0.97
D8	5.5	6.0	6.0	4.0	5.33	0.94
D9	6.5	7.0	4.0	3.5	4.83	1.55
D10	6.0	5.0	4.0	3.0	4.00	0.82

### Material collection and loss rate

**Fig. 18:**
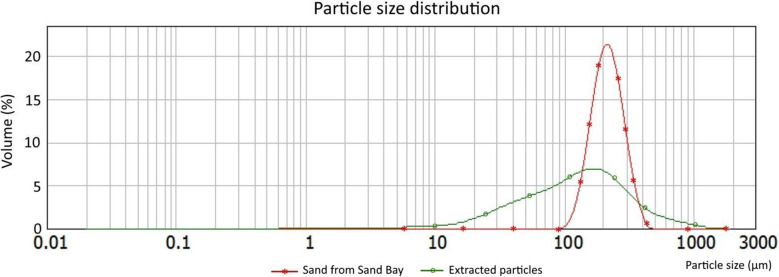
Particle size distribution of material extracted from M1-N and unmodified sand from Sand Bay

Marimo M1-N was swirled in a dish with 25.040 g of beach sand and deionised water for 20 h. A comparison of the particle size distribution (measured with Mastersizer 2000) of the material collected by M1-N and extracted by subjecting it to centrifugal rotation (1 h) against the original beach sand is shown in Fig. 18 (see ESI S2 for additional details).

To explore possible loss of biological material during the above experiment, a separate Marimo (M1-O) was similarly swirled in an empty dish for 20 h. The change of dry weight of both dishes, material collection, and material removal from Marimo are shown in Table 3. The weight of material collected by M1-N (334.1 mg) was far greater than the material lost by M1-O (~0.3 mg). The total mass of material obtained by subjecting it to centrifugal rotation (460 mg) originated from a combination of sand collected from the dish (334.1 mg), possibly some previously trapped material released, and organic material (substantially Marimo algal filaments) loosened by agitation against the sand bed. The mass of inorganic material (301.1 mg) determined using the previously described method was approximately twice as large as organic material (158.9 mg).

The mass of extracted inorganic material (~301 mg) was less than the mass of sand collected from the bed (~334 mg) which indicated the Marimo may still contain ~33 mg, and centrifugal rotation is ~90% efficient. The minimal mass of matter added to the empty dish (~0.3 mg) suggests that moving across a smooth surface (in this case, glass) reduced material loss compared to moving across a “rough” sand bed for the same period (158.9 mg). In other words, the rapid movement of Marimo across a rough (sand) surface causes pieces of filamentous algae to detach.

**Table 3:** Rates of material collection and loss from Marimo subjected to centrifugal rotation

Marimo (reference)	Rotation time (h)	Initial sediment (g)	Swirl time (h)	Remaining dish weight (g)	Change dish weight (g)	Rotation time (h)	Extracted inorganic (g)	Extracted organic (g)
*M1-N*	1	25.040	20	24.7059	− 0.3341	1	0.3011	0.1589
*M1-O*	1	0	20	0.0003	+ 0.0003	1	0	0

### Marimo filtration

A range of standard biological and physicochemical water quality indicators is shown in Table 4. These recordings were taken before and after circulation through the Marimo filter and a control filter. After 24 h of circulation, significant reductions in indicator species were observed. For *E. coli*, a reduction of 98.6 ± 0.82% was observed in the Marimo circulated sample, while an increase of 44.3 ± 32.4% was observed in the control (both from a starting density of 657 ± 600 CFU 100 mL^−1^. Similar results were observed for enterococci, where a reduction of 98.5 ± 1.97% was observed in the water circulated through the Marimo filter in comparison to a 20.6 ± 12.7% reduction in the control system. There was a significant reduction in turbidity from 34.3 ± 10.5 to 7.33 ± 2.49 NTU when circulated through the Marimo filter, in comparison to 32.3 ± 10.8 NTU in the control. For nutrient analysis, there was a significant reduction in total inorganic carbon (TIC) of 34.3 ± 13.6% from 7.50 ± 0.86 to 5.04 ± 1.50 in the Marimo system. However, no significant differences were observed for the other nutrients measured. There were significant decreases in both total dissolved solids (TDS) and conductivity observed in the Marimo system, in comparison to the control system. There were no significant differences observed for the remaining parameters measured.

**Table 4:** Water quality parameters of environmental water pre-circulation (effectively fresh water), post-control (water circulated through an empty filter column with no Marimo for 24 h), and post-Marimo (water circulated through the system with a Marimo filter for 24 h) (= 3). *p*-values compare post-control vs post-Marimo (unpaired *t*-test, two-tailed). * = < 0.05

Parameter	Pre-circulation	Post-control	Post-Marimo	*p*-value
Presumptive *E. coli* (CFU/100 mL)	657 ± 600	642 ± 482	14 ± 17	0.087
Enterococci (CFU/100 mL)	933 ± 831	1117 ± 1150	30 ± 42	0.177
Total organic carbon (mg/L)	1*.*30 ± 0*.*10	1*.*47 ± 0*.*08	1*.*23 ± 0*.*08	0.021 *
Total inorganic carbon (mg/L)	7*.*50 ± 0*.*86	7*.*37 ± 0*.*82	5*.*04 ± 1*.*50	0.078
Ammonium (mg/L)	0*.*06 ± 0*.*03	0*.*05 ± 0*.*03	0*.*03 ± 0*.*03	0.460
Nitrite (mg/L)	0*.*61 ± 0*.*21	0*.*63 ± 0*.*21	0*.*59 ± 0*.*20	0.823
Nitrate (mg/L)	6*.*43 ± 4*.*53	6*.*70 ± 4*.*50	5*.*63 ± 4*.*21	0.779
Phosphate (mg/L)	1*.*81 ± 0*.*77	1*.*85 ± 0*.*66	0*.*99 ± 1*.*07	0.302
Dissolved oxygen (mg/L)	6*.*53 ± 1*.*00	7*.*15 ± 0*.*46	8*.*04 ± 0*.*92	0.208
Oxidation–reduction potential (mV)	315 ± 66*.*2	296 ± 37*.*5	291 ± 34*.*6	0.874
Potential of hydrogen (pH)	7*.*83 ± 0*.*60	8*.*15 ± 0*.*06	8*.*44 ± 0*.*34	0.219
Conductivity (S/cm)	839 ± 19*.*8	810 ± 33*.*6	735 ± 51*.*8	0.103
Total dissolved solids (mg/L)	574 ± 14*.*8	559 ± 15*.*5	497 ± 33*.*5	0.044 *
Turbidity (NTU)	34*.*3 ± 10*.*5	32*.*3 ± 10*.*8	7*.*33 ± 2*.*49	0.018 *

**Fig. 19:**
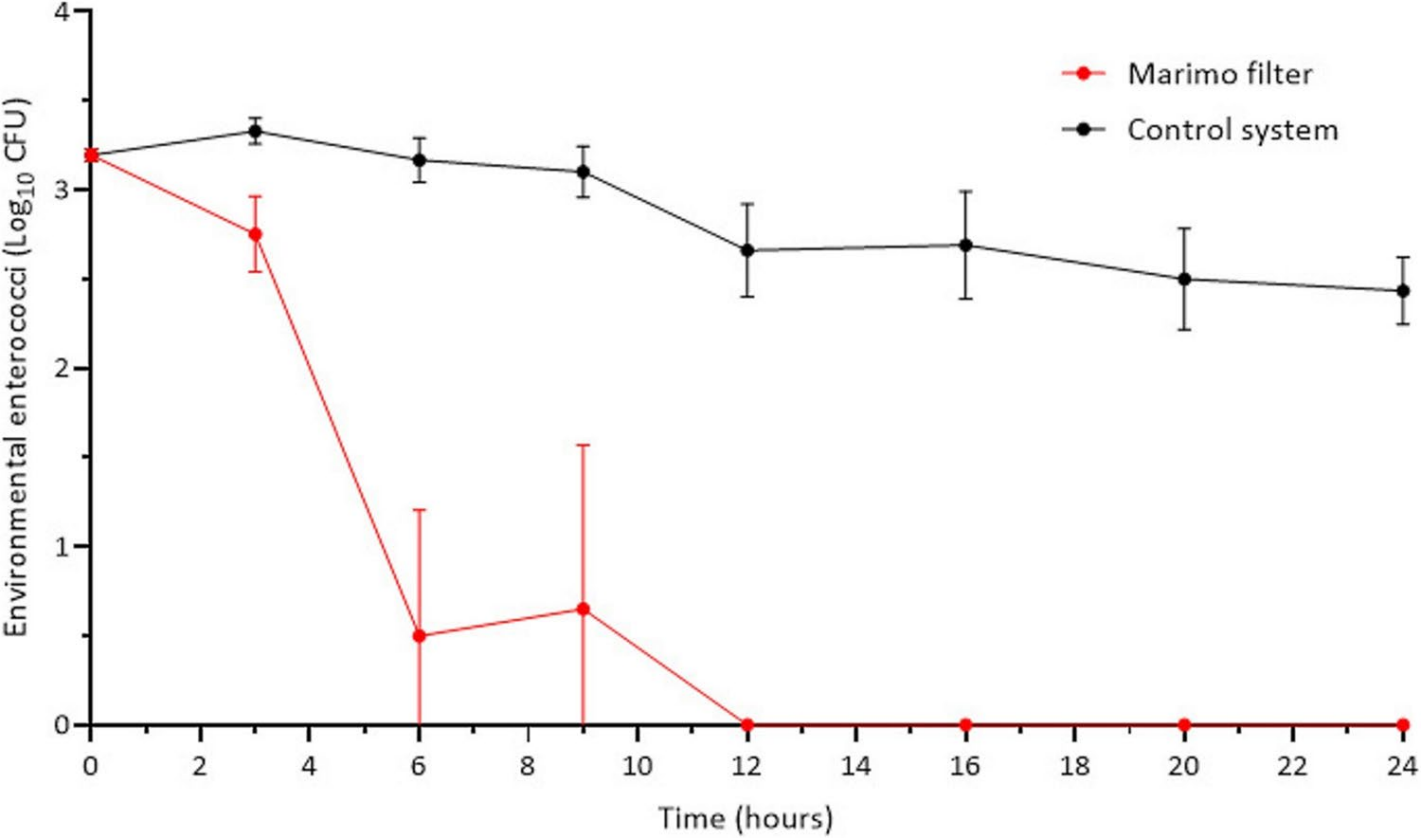
The total number of indicator species, enterococci, in the environmentally derived surface water whilst being recirculated through the Marimo filter for 24 h

To better understand the effectiveness of the system, the level of enterococci within the environmental water was monitored at regular intervals for one of the experimental runs. This is presented in Fig. 19. The Marimo filter was able to reduce the levels of enterococci (starting density of 157 CFU 100 mL^−1^) within approximately 12 h. Moreover, this demonstrates that the level of enterococci was relatively stable over the experimental period: 3.19 Log_10_CFU at the start of the experimental run and 2.43 Log_10_CFU after 24 h in comparison to 0 Log_10_CFU when cycled through the Marimo filter.

### RFID tagging

Due to the importance of Marimo being able to roll and periodically float, the effects of radio-frequency identification (RFID) tagging were investigated. The insertion of the RFID tag was performed using a needle at the centre of the Marimo. Insertion was quick and easy, with no signs of the RFID tag being able to come out. It was observed that the Marimo with the RFID tag in the centre continued to float in the presence of light (see Fig. 8c). Additionally, no noticeable change in Marimo rotation or centre of balance was observed. Continued observation over a 6-month period revealed no visible impacts on the health or behaviour of the tagged Marimo.

## Discussion

A comprehensive analysis of the findings, offering insights into the potential of Marimo as a tool for environmental monitoring and filtration in aquatic environments, is presented in this section.

### Extracting environmental samples from Marimo

A novel, low-cost, and environmentally friendly method for sampling aquatic environments using Marimo has been demonstrated. The method relies on applying a low relative centrifugal force (RCF) of approximately ~1 g, which is sufficient to extract entrapped materials without visibly harming the Marimo.

RCF depends on the speed of rotation (e.g. 200 rev/min) and the distance of the artefact from the centre of rotation (e.g. 25 mm). By maintaining a low RCF of ~1 g, it was possible to remove artefacts without any apparent harm to the Marimo. If the speed of rotation is increased (for example, placing Marimo in a high-speed vortex flow generated by pumped current flow(s) rather than rotation flow), the extraction time might be reduced.

However, prolonged rotation (e.g. ~100 h) led to significant organic mass loss, likely due to filament abrasion. Therefore, further studies are needed to determine the optimal rotation time that balances extraction efficiency with minimal damage.

To further isolate Marimo-specific effects from those induced by fluid dynamics alone, future studies should include control trials where magnetic stirrers are rotated in similar environments without Marimo present. This would allow researchers to quantify background sediment mobilisation and artefact displacement attributable solely to the rotating fluid. Such controls would help clarify the extent to which observed extraction and filtration effects are due to Marimo’s unique filamentous structure, rather than mechanical agitation alone.

Destructive analysis of the “Whole” Marimo (i.e. without rotation, see “Gravity sedimentation and organic dissolution of extracted material”) revealed that rotation extracts only a portion of the trapped inorganic material. This is supported by optical and X-ray imaging, which shows residual artefacts deep within the Marimo, likely deposited during earlier growth stages.

The behaviour of Marimo in rotating fluids, where they migrate toward the centre and achieve stable rotation, is consistent with established fluid dynamics principles (van Kuik et al. (2015); Okulov et al. (2015); Einstein (1926); Hopfinger and Van Heijst (1993)). This behaviour is driven by pressure gradients and drag forces within the rotating medium. In essence, rotation creates a differential pressure gradient in the water, with the higher pressure near the outer boundary (e.g. wall of the beaker) and the lower pressure in the middle. The inward force on the object near the bottom is the same as the inward force felt at any other depth. Therefore, the acceleration of the filaments towards the centre will be the same as well. However, the velocities will not be the same due to drag. The velocity near the bottom of the beaker will be lower due to increased drag. When the velocity is not sufficient to maintain an orbit, the filaments will migrate towards the centre of the beaker. This creates a secondary flow of fluid towards the centre. This inward fluid flow causes the Marimo to move towards the centre of the beaker. For stable rotation of Marimo, several parameters (listed in “Extracting environmental samples from Marimo”) need to be within specific ranges.

Overall, Marimo-based sampling offers several advantages: minimal environmental impact, the ability to capture a wide range of pollutants, and the potential for automated analysis using machine vision and artificial intelligence. Future work could explore optimising extraction parameters and integrating RFID tracking for spatial monitoring.

A detailed study of the material removal rate against time (while rotating) was not conducted as it is outside the scope of this work.

### Optical inspection of Marimo ball

**Fig. 20:**
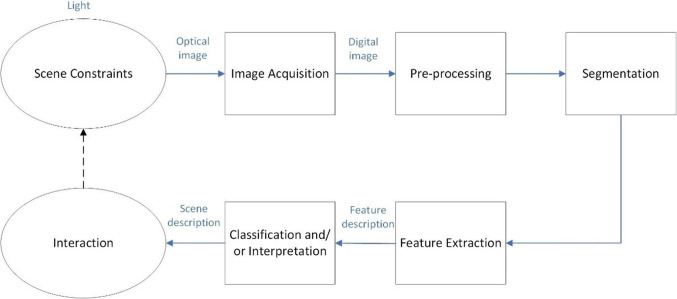
Five stages of classical machine vision.

The application of digital image processing tools and machine learning algorithms is now indispensable in scientific and engineering visual inspection tasks, for example, medicine (Wimmer et al. (2021)). The richness of data afforded by image files facilitates the implementation of image processing techniques ranging from traditional basic particle analysis methods to the aforementioned intelligent analysis techniques. Classical machine vision can be broken down into five essential stages (Smith et al. (2021), see Fig. 20). The first stage is often considered the most important, where environmental structuring is paramount. In this and in most tasks, perhaps the most important stage is image acquisition. Scene constraints, lighting, and object positioning significantly affect image acquisition. At this early stage, achieving the best image has a significant beneficial effect on subsequent processing. The spherical nature of Marimo makes it a difficult object to image, as a single sensor is unable to capture the whole surface. In this case, only partial surface imaging can be achieved using a traditional imaging system: a single camera and lighting. Therefore, an assumption must be made that the captured portion of the surface is proportionally representative of the whole surface. As a result, typically, this method suffers from inaccuracy. An alternative method is to displace the particles that are being measured from the sphere and present them on a planar surface for simplified imaging. Here, it is assumed that all the particles have been successfully displaced and presented to the vision system. However, this method also has reliability issues but can be more representative of the artefacts on the Marimo. Moving on to the next stage, pre-processing, the job is to reduce noise and, if possible, make more obvious those particles that are being measured. Once this is achieved, then differentiating between good and bad data is the task; the particles being measured or counted need to be separated from the background. With the current application in mind, the first three stages of machine vision are most relevant. Ultraviolet lighting has been used to enhance the detail of the scene. The artefacts have been displaced from the Marimo and presented on a planar surface for the computer to then process once the images have been captured.

With the application of well-established and simple particle analysis techniques, the majority of particles can be counted. These can be further sifted by inputting data relevant to the size and shape of the particle, expected to improve results. Even then, there may be some errors where particles may either fully or partially overlap. Future work will consider more complex techniques, such as multi-scale tracing of curvilinear features [38] and shape from shading [37], which could be investigated to ensure more robust segmentation.

Although the two Marimo compared were different, the greater number of artefacts (approximately double the number counted) and noticeably larger physical size of said artefacts indicate that being subjected to centrifugal rotation for ~1 h is effective at removing some (but not all) particles. The use of two different Marimo reduces variation in setup but introduces variation in measurements.

Artefact counting via ultraviolet black light inspection was found to be sensitive to setup. In other experiments (not reported here), optical inspection of the same Marimo before and after rotation was trialled. However, positioning the Marimo and/or camera to photograph the same section of the Marimo after rotation is challenging (due to the Marimo’s spherical shape, lack of orienting feature(s), and movement of algal filaments). Possible solutions include the following: (i) scanning the entire (rather than a section) of the surface before and after rotating and comparing images with bespoke software; (ii) adding a temporary visual marker on the surface for re-orientation after rotation; (iii) assuming the collection rate is close to uniform across the surface and comparing different sections.

To simplify the task, the artefacts were imaged on a planar surface after being displaced from the rotating Marimo. The environment was structured with ultraviolet black lighting to best reveal the particles. A proprietary software tool (Photoshop 2022, version 23.3.2, Adobe Systems Inc., USA) was used to count the prevalent particles. This was essentially a particle analysis task.

Further study (involving end users) is suggested to gain a clear understanding of the most beneficial technique for large-scale environmental monitoring.

### Analysis of extracted material

Table 1 reveals considerable variability in the material obtained from Marimo of similar size, sourced from the same supplier. In particular, the total mass of debris extracted by subjecting it to centrifugal rotation (immediate on possession, i.e. no particles added by us) for ~1 h ranges from 0.180 to 1.090 g. The mass of inorganic matter extracted varies from 0.065 to 0.500 g. This suggests that some of the Marimo have had greater contact with sediment than others. It is worth noting that a small number of larger (heavier) particles could bias the results of a large number of smaller (much lighter) particles. The mass of organic matter extracted also varies considerably from 0.050 to 0.590 g, suggesting that filaments on some Marimo may have been less securely attached, dead filaments, or that the Marimo have collected organic matter (which was dissolved away during processing; see “Gravity sedimentation and organic dissolution of extracted material”). For interest, the organic matter of a Marimo was also entirely dissolved (Marimo and organic sediment), and the remaining inorganic mass was 1.113 g.

The original source of the acquired Marimo is believed to be Lake Svityaz in Ukraine (Boedeker et al. (2010)). At 58.4 m deep and with a surface area of 25.2 km^2^, it is one of the largest lakes in Ukraine (Schworer et al. (2022)). However, both this origin point and possible Marimo handling prior to purchase could not be confirmed. Microscope images of extracted particles are consistent with the reported makeup of the lake bed of Lake Svityaz (Zhabina et al. (2017); Schworer et al. (2022); Ilyina and Ilyin (2014)). For example, “gray lake-marsh silty and sandy muds with coalified phytodetritus” (Zhabina et al. (2017)) can be observed in Fig. 10b, and “Gray calcareous sandstones with organogenic detritus transformed into bioclastic sandy limestones with oolites” (Zhabina et al. (2017)) can be observed in Fig. 10.

As this study represents early-stage, exploratory research, our resources were limited and did not permit multiple repeats under identical conditions for each Marimo specimen. Consequently, statistical reproducibility across specimens and sizes was not formally assessed in this initial phase. However, the observed variability in extracted material (as shown in Table 1) provides valuable insight into the potential of Marimo to accumulate environmental debris. These preliminary findings justify further investigation, including controlled, repeated trials to rigorously assess inter-sample variability and reproducibility.

### Particle size distribution of inorganic residue

Images appear to indicate that the inorganic material extracted is likely composed of quartz and calcite grains across a range of sizes (Fig. 10). The size distribution of particles extracted by rotation (Fig. 11) confirms that the majority of samples are dominated by silt particles, peaking at ~50 µm while the particles obtained by dissolution of a whole Marimo peaks at ~300 µm, indicating a medium sand. Sample M1-C, however, shows a bimodal distribution due to the presence of medium/coarse sand grains at ~50 µm and ~750 µm. Sand grains appeared well-rounded (Fig. 10c).

A comparison of particle size distributions of extracted inorganic particles from Marimo (Fig. 11) indicates the following: three samples M1-A, M1-B, and M100-A (extraction by rotation) have similar particle size distributions with predominant extracted particle size ~40 µm (by percentage volume); sample M1-C (extracted by rotation) has a second peak ~800 µm possibly due to collecting sediment from a different locality; and the particle size distribution of a wholly dissolved Marimo indicates that larger particles (peak ~300 µm) may be unable to escape the centre of the filament ball during centrifugal rotation for 1 h.

### Microplastic analysis

#### Analysis by optical spectroscopy

It was observed (under ultraviolet illumination) that some fibres had fluorescence properties, while others did not. For example, the black fibres in Fig. 12c do not fluoresce.

#### Analysis by Raman spectroscopy

Of the seven fibres identified, four were identified as microplastics. Fibres 2 and 5 were polyethylene terephthalate (PET), and Fibres 6 and 7 were polypropylene (PP). The other three fibres had a rough ribbon-like appearance and were probably natural fibres (cellulose). Fibre 4’s spectrum also indicated the fibre was cellulose. The two probable cellulose fibres appeared encrusted with crystalline particles and gave spectra for calcite. It is possible that the cellulose fibres became calcified with age. The calcium carbonate encrustations may have protected the organic fibres during the digestion method.

In summary, Raman measurements confirm that a range of microplastics are captured and retained by Marimo. These measurements support published findings by other researchers (Boedeker et al. (2010)). Furthermore, these measurements confirm that microplastics can be extracted from Marimo by centrifugal rotation.

#### Analysis by FTIR spectroscopy

Table S4 in ESI shows that Marimo is capable of capturing a diverse range of microplastics from the environment. For example, extracted material included fibres (of various colours), fragments, and dots. Figure 15 shows a clear polyester fibre (~15 µm diameter, ~500 µm length) extracted from Marimo sample M1-V. The high Hit Quality Index (HQI) of 0.96 indicates a high confidence level in identification. Fig. S10 in ESI shows a white Polyvinyl Chloride (PVC) fibre (~40 µm diameter, ~700 µm length) extracted from Marimo sample M1-W. HQI of 0.71. Fig. S11 in ESI shows a light brown Polyvinyl Chloride (PVC) fragment (~70 µm diameter, ~200 µm length) extracted from Marimo sample M1-X. HQI of 0.63. No plastics were identified in the mapped areas of the three samples (M1-V, M1-W, M1,-X); however, quartz and cellulose particles were abundant (see Fig. 16).

### Micro-organism analysis

Processing with hydrogen peroxide removes the majority of the organic material, leaving only the traces of a few microorganism species. Representative examples of siliceous structures include the non-organic silica frustules of diatoms (Fuhrmann et al., 2004; Bradbury, 2004; Lopez et al., 2005) and robust testate amoebae such as *Cyphoderia ampulla*, which possess siliceous scales encased in a protective layer of hard organic cement (Todorov et al., 2009). It is likely that other, more delicate groups of microorganisms gathered by or living on the Marimo were degraded by the hydrogen peroxide prior to examination. Consequently, future studies should consider using more focused, non-destructive extraction techniques to optimise the isolation of particular microbial groups of interest.

### Environmental sustainability

Visual inspection of Marimo and measurement of the rate of gas generation after centrifugal rotation for 1 h indicate that extracting material does not significantly harm Marimo’s health. Conversely, removing debris and dead organic material may improve their growth potential and possibly extend their life. Longer-term studies are required to confirm these initial findings. Lake environments are complex, with many interacting factors requiring detailed consideration and monitoring (Wakana et al. (2021); Oyama et al. (2016)).

While gas production was monitored post-centrifugation as a proxy for Marimo health, we acknowledge that this metric alone may not fully capture physiological stress. Parameters such as chlorophyll content and photosynthetic efficiency are widely recognised indicators of algal health and stress response (Grzesiuk et al. (2022)), and we propose their inclusion in future work to provide a more comprehensive assessment of Marimo viability following mechanical treatment.

To better understand the long-term viability of Marimo as a filtration medium, future studies should investigate fouling dynamics, biomass turnover, and regeneration strategies under continuous operation. Specifically, experiments should be designed to quantify the rate and nature of biofouling on Marimo surfaces, assess the degradation or shedding of algal filaments over time, and evaluate the potential for structural and functional recovery following cleaning or rest periods. These data would inform estimates of operational lifespan, maintenance intervals, and ecological resilience, thereby supporting the development of sustainable Marimo-based filtration systems for real-world deployment.

The attenuation coefficient of pure water, averaged across the photosynthetically active radiation (PAR) spectrum, is approximately 0.03 m⁻¹ (Pedersen et al., 2013). The deepest that light can penetrate water with sufficient intensity for photosynthesis depends on water clarity (Abdelrhman (2016); Fukushima et al. (2016)). For example, plant growth at a depth of 70 m with just 10% of surface irradiance being available has been reported (Duarte (1991)). Therefore, freshwater lakes and watercourses of modest depth (~10 m) might be suitable for Marimo-based environmental monitoring. Other organisms might be suitable for sustainable environmental sampling in other environments. Care needs to be taken to avoid introducing alien species into local environments. For example, the invasive spread of *Dreissena polymorpha* (zebra mussels) (Strayer (2009); Nalepa and Schloesser (1992); Bossenbroek et al. (2007); Molloy et al. (1997); Zaiko et al. (2009)) is of particular concern as they can be carried on Marimo (Patoka and Patoková (2021); Mull and Spears (2022)). The possibility of removing invasive species (such as zebra mussels) from Marimo without harm via centrifugal rotation might have useful applications but is outside the scope of this study.

To minimise the potential environmental impact of the research, only 30 Marimo of modest physical size (~50 mm diameter) were purchased and reused where possible. Further, research into “creating” Marimo in the laboratory by growing and intertwining algal filaments (to avoid removing Marimo from nature) is being conducted and will be reported separately. “Tangled-type” (Nakai et al. (2021)) Marimo may have different properties with regard to collecting, retaining, and releasing material than the “radial type” studied as part of this research.

Optimal water temperature for Marimo to grow is reported to be 5 to 22 °C (Nakayama et al. (2023)) which predominantly corresponds with geographical locations in northern latitudes. Suitable regions include Japan, Iceland, Austria, Sweden, and Estonia (Nakayama et al. (2023)).

### Material collection and loss rate

A comparison of the particle size distribution of inorganic material collected by and extracted from M1-N compared to the initial field sample (fine sand from Sand Bay) is shown in Fig. 18. The field sample had a well-sorted distribution with a mean particle size of 224 µm (median 216 µm). Following the experimental treatment described (“Material collection and loss rate”), the inorganic material extracted from the Marimo presented a broader, less well-sorted particle size distribution with a mean of 177 µm (median 128 µm). These initial findings appear to indicate that Marimo pick up and retain sand which is then preferentially extracted and/or larger particles are preferentially retained, based on the experimental conditions that the Marimo were subjected to (swirled on an orbital shaker at 50 rev/min for 24 h). Further, the volumetric distribution of collected and extracted particles is biased towards smaller diameters. These two factors might indicate that Marimo is more efficient at collecting smaller (i.e. sub 200 µm) rather than larger particles.

Details of both material collection and loss from the artificial sediment bed to Marimo are shown in Table 3. Data show that M1-N picked up sand (334.1 mg), and despite centrifugal rotation for 1 h to extract material, it appears that some material (~ 33 mg) was retained. Additionally, organic material (probably loose algae filaments) was extracted (~ 159 mg). Over the same period, sample M1-O only lost ~ 0.3 mg. The rate of material gain is three orders of magnitude greater than the rate of material loss.

A more detailed study of material collection, retention, and extraction (outside the scope of this study) could provide further clarity. For example, centrifugal rotation at higher speeds might enable particles with larger diameters to be extracted. Further, mapping particle diameter against rotation speed might facilitate low-cost particle size analysis.

### Marimo filtration

There was a significant reduction in biological indicator species after 24 h of circulation through the Marimo filter.

The starting density of both indicator species was highly variable (150–1500 CFU 100 mL^−1^ for *E. coli* and 230–2100 CFU 100 mL^−1^ for enterococci), due to the highly variable nature of the environmental water used in this study that exhibits a high throughput of runoff from the surrounding area. Nonetheless, the Marimo filter was able to significantly reduce the presence of the indicator species within the reservoir of the filter system. The use of biofilms established in filter columns has been shown to reduce waterborne pathogens (Steven et al. (2022)). This work suggests that the presence of an active biofilm was the result of the reduction in pathogenic bacteria present in freshwater. This could explain the reduction observed using the Marimo filters, the production of antibacterial compounds by the Marimo, and/or the bacterial biofilms established within the Marimo that resulted in the reduction of the indicator species. Moreover, the significant reductions observed in turbidity and TDS demonstrate that the Marimo were able to capture and remove particulates in this configuration. This may have resulted in the capture of the indicator species. Decreases in TIC were likely caused by the consumption of CO_2_ through photosynthesis, which may also be suggested by a non-significant increase in DO.

A practical application of this work could use the recirculating Marimo systems, in series, to reduce the biological burden of contaminated water discharges with high bacterial loads that could potentially help improve the water quality of inland freshwater systems. Moreover, the deployment of novel biological treatment systems incorporating Marimo could help improve the quality of water used in aquaculture where maintenance of water quality is critical (Defoirdt et al. (2011)).

### RFID

To infer the geolocation of the collection path/area, an RFID tag (Husár et al. (2020); Rayhana et al. (2021)) can be inserted into the centre of the Marimo. For example, tags compliant with ISO11784/5 usually have a microchip which provides 8 Startbits and 14 “Reserved for future use” bits (which are commonly used to tag domestic pets). This enables the locations and times of release and capture to be cross-referenced and integrated with other data (such as local currents).

Because RFID tags need to be centrally placed for buoyancy purposes, the maximum size Marimo that can be tagged is limited by the operating range of said tag. The rated operating range of a particular RFID scanner depends on the standard of the tag and the model of the scanner. For example, FJ-380 (Fonkan, China) with an ISO 11784/5 FDX-B tag has a rated range of 80 mm, which exceeds a radius of 25 mm and permits tagging of Marimo up to a diameter of 160 mm.

RFID tagging combined with a “catch and release” policy could be used to both monitor the lifetime pathways of Marimo, as well as monitor the currents that form them. Marimo can either be collected manually by humans or the process of capture might be automated with tethered robotic platforms or an autonomous underwater vehicle (AUV) (Monk et al. (2018); Mondal and Banerjee (2019); Yamahara et al. (2019)).

### Future work

While this study has demonstrated the potential of Marimo as a passive, eco-friendly tool for aquatic environmental monitoring and filtration, several avenues remain for future exploration. One important direction is the comparative evaluation of Marimo-based filtration against other natural or engineered filtration systems. These include biofilms, aquatic macrophytes (e.g. duckweed, water hyacinth), and sponge filters, which have been widely studied for their ability to remove contaminants from water. Each system offers distinct advantages and limitations in terms of selectivity, retention time, scalability, and ecological compatibility.

Although a direct head-to-head benchmarking study was beyond the scope of this initial investigation, we acknowledge the importance of such comparisons and intend to pursue them in future work. Preliminary observations indicate that Marimo offers several benefits, including the potential for cleaning via centrifugal rotation, passive operation, minimal infrastructure requirements, and suitability for in situ deployment in freshwater environments. Future studies will also explore: repeated trials to rigorously assess inter-sample variability and reproducibility; controlled field experiments with synthetic microplastics to quantify Marimo’s filtration efficiency under real-world conditions; long-term performance and fouling behaviour under continuous operation; physiological assessments including chlorophyll content and photosynthetic efficiency post-filtration; control trials where magnetic stirrers are rotated without Marimo present; assess fouling, biomass turnover, and regeneration strategies to better understand the long-term viability as a filtration medium; and integration with autonomous monitoring platforms for scalable deployment.

These efforts will help establish Marimo’s role within the broader landscape of sustainable water monitoring and treatment technologies and support its potential application in aquaculture, environmental remediation, and citizen science.

## Conclusions

This study has demonstrated that Marimo (*Aegagropila linnaei*) can effectively capture and retain a diverse range of environmental contaminants, including microplastics, sediments, and microorganisms such as *E. coli* and enterococci. The use of Marimo offers several advantages over traditional sampling methods, including the ability to collect materials passively over extended periods, minimal ecological disruption, and compatibility with environments where Marimo naturally occur. Analytical techniques such as optical microscopy, Raman spectroscopy, and FTIR were successfully employed to characterise the extracted materials, with each method contributing unique insights into the composition and diversity of the captured particles. The study also introduced a Marimo-based filtration system that achieved a significant reduction in microbial contaminants, highlighting its potential for applications in water treatment and aquaculture. Despite these promising results, limitations remain, including the inability to precisely determine the sampling location without RFID tagging and the challenge of extracting all entrapped material without compromising the integrity of the Marimo. Nonetheless, the findings suggest that with further development (particularly in areas such as automated analysis, optimised extraction protocols, and scalable deployment), Marimo could serve as an effective and environmentally friendly solution for long-term aquatic ecosystem monitoring and remediation.

## Supplementary Information

Below is the link to the electronic supplementary material.

Supplementary file1 (PDF 4428 KB)

## Data Availability

The datasets generated and analysed during this study are available from the corresponding author upon reasonable request.
