# Supplementary material for: Marimo for monitoring and filtering of aquatic environments
Source: Environ Sci Pollut Res Int. 2025 Dec 19;32(56):30808–32. doi: 10.1007/s11356-025-37259-6 (PMC12804339; doi:10.1007/s11356-025-37259-6)
Supplement: Supplementary file 1 — Supplementary file1 (PDF 4428 KB) [file 11356_2025_37259_MOESM1_ESM.pdf]

Electronic Supplementary Information (ESI)

Marimo for monitoring and filtering of aquatic environments

Neil Phillips[*,a], Thomas C. Draper[b,a], Andrew P. Geary[c], Kathryn Lamb-Riddell[b], Darren M. Reynolds[b,d], Joshua A. C. Steven[d], Freya Radford[e], Abdul R. Farooq[f], and Andrew Adamatzky[a]

[a]Unconventional Computing Laboratory, University of the West of England, Bristol, UK
[b]Institute of Bio-Sensing Technology, University of the West of England, Bristol, UK
[c]School of Architecture and Environment, University of the West of England, Bristol, UK
[d]Centre for Research in Biosciences, University of the West of England, Bristol, UK
[e]School of Applied Sciences, University of the West of England, Bristol, UK
[f]Centre for Machine Vision, University of the West of England, Bristol, UK
[*]Corresponding author, email: neil.phillips@uwe.ac.uk

S1 Particle size distribution from gravity sedimentation

Particle size distribution of inorganic residue obtained from Marimo by gravity sedimentation plotted in Fig. S1.

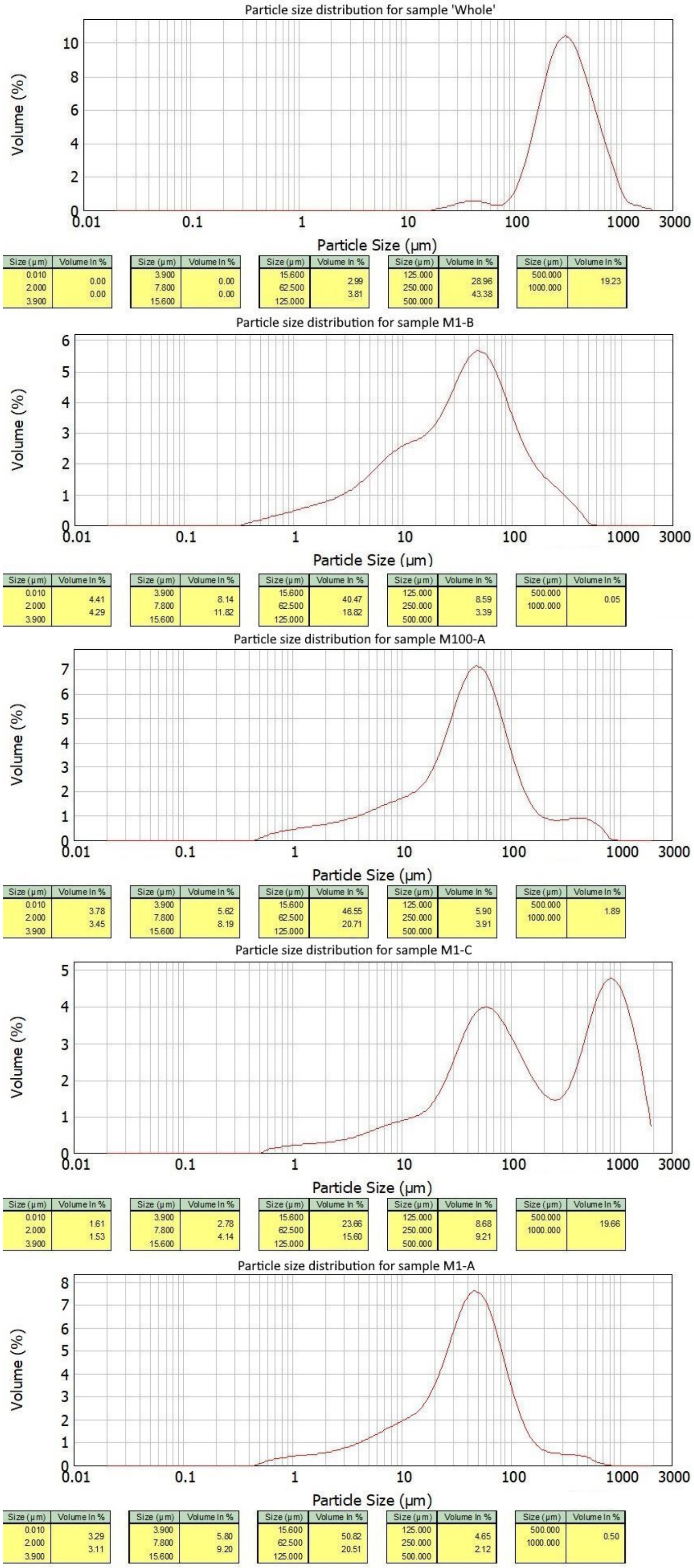

Figure S1: Particle size distribution from gravity sedimentation

S2 Particle size distribution from collection and loss rate measurements

Particle size distribution of inorganic residue plotted in Fig. S2.

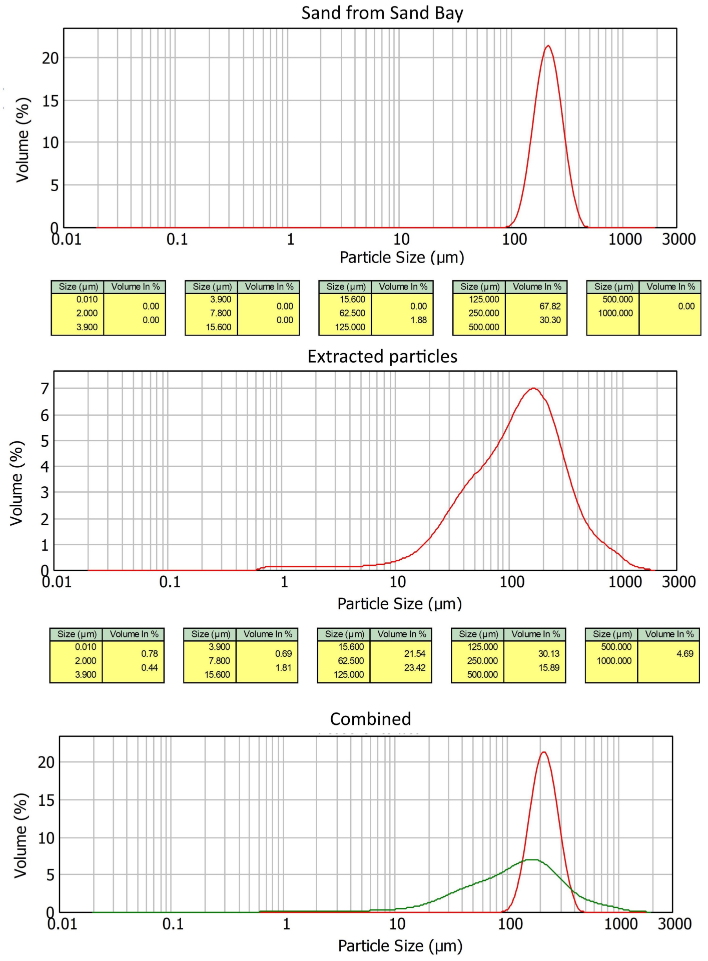

Figure S2: Particle size distribution from collection and loss rate measurements

Details of the particle size distribution of inorganic residue are shown in Table S1 and S2.

Table S1: Particle size distribution

	Volume weighted mean D [4, 3]	Surface weighted mean D [3, 2]	Median d (0.1)	Median d (0.5)	Median d (0.9)
Sand	223.378	207.392	151.004	215.307	306.99
Sand	224.679	208.611	151.955	216.534	308.681
Sand	224.353	208.454	152.005	216.38	307.785
Sand (average)	224.137	208.151	151.648	216.073	307.824
Extracted	184.144	44.28	30.475	129.259	376.874
Extracted	172.224	43.916	30.404	127.567	358.437
Extracted	173.823	44.528	30.795	129.941	370.355
Extracted (average)	176.73	44.24	30.556	128.917	368.226

Table S2: Particle size distribution

Sizes in µm	2	3.9	7.8	15.6	62.5	125	250	500	1000
Sand	0	0	0	0	0	1.9714	68.1309	29.8975	0
Sand	0	0	0	0	0	1.8346	67.5709	30.5943	0
Sand	0	0	0	0	0	1.8224	67.7634	30.4140	0
Sand (av)	0	0	0	0	0	1.8761	67.8218	30.3019	0
Extracted	0.78450	0.43905	0.69313	1.83362	21.49413	23.3360	30.1309	15.2223	4.7862
Extracted	0.79112	0.44622	0.69764	1.83329	21.78457	23.5891	30.2507	15.7858	4.4325
Extracted	0.77916	0.44578	0.68971	1.77667	21.34689	23.3301	30.0180	16.6532	4.8573
Extracted (av)	0.78493	0.44368	0.69349	1.81453	21.54186	23.4184	30.1332	15.8871	4.6850

S3 Additional Raman Results

Table S3: Raman spectroscopy results for fibres and particle extracted from Marimo

**Sample**	**Description**	**Peaks (cm^{-1})**	**KnowItAll Database ID Match**
1	Fibre, ribbon-like fibre 50 µm to 80 µm diameter encrusted with crystalline particles.	281.45, 712.81, 1085.93	Calcite 84.87%<br>Probably cellulose
2	Fibre, single, thin, smooth fibre. Colourless fibre, 10 µm, encrusted with rough particles.	279.41, 631.66, 702.78, 858.00, 997.81, 1093.84, 1289.63, 1417.21, 1614.45, 1726.28	Polyethylene terephthalate (PET) 85.03%
3	Fibre, single, thin, rough fibre.	278.67, 712.27, 1085.64	Calcite 81.88%
4	Fibre, single, irregular, rough fibre, 50 µm to 100 µm diameter	380.12, 808.15, 1094.43, 1122.13, 1453.42	Cellulose 74.30%
5	Fibre, single, thick, smooth fibre, 50 µm to 60 µm diameter.	631.98, 702.39, 794.46, 858.31, 997.81, 1093.26, 1291.60, 1416.66, 1614.19, 1726.28	Polyethylene terephthalate (PET) 82.46%
6	Fibre, thin fibre cluster 10 µm to 20 µm diameter.	399.45, 808.77, 840.64, 972.53, 998.11, 1044.05, 1152.26, 1167.41, 1220.55, 1328.86, 1358.02, 1457.51	Polypropylene 77.03%
7	Fibre, thin, irregular fibre.	399.11, 809.08, 841.57, 896.33, 943.44, 973.14, 1035.44, 1150.21, 1167.12, 1217.68, 1329.14, 1359.96, 1459.97	Polypropylene 73.97%
8	Particles, numerous irregular, shiny, particles of 20 µm to 40 µm diameter.	None, very poor spectra only, no significant difference from the background substrate	No definitive ID

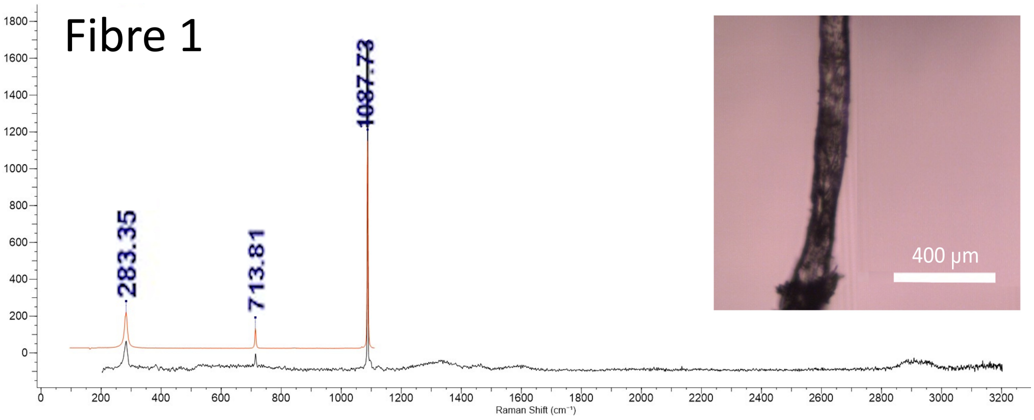

Figure S3: **Fibre 1 Raman spectrum:** Raman spectrum (black), and database spectrum (red). Sharp peaks at 283.35, 713.81, 1087.73 cm^{-1}. KnowItAll database ID match **Calcite 84.87%.** Rough, ribbon like, appearance suggests this is a natural (cellulose) fibre.

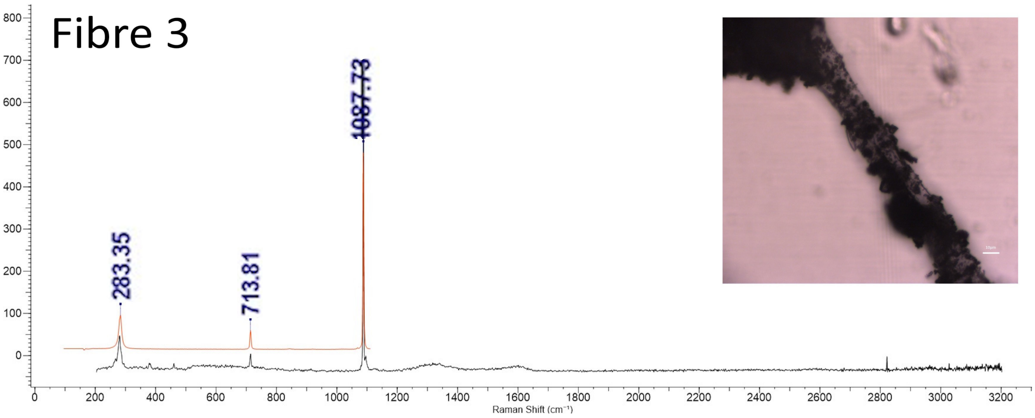

Figure S4: **Fibre 3 Raman spectrum:** Raman spectrum (black), and database PP spectrum (red). Raman spectrum: Sharp peaks at 278.67, 712.27, 1085.64 cm^{-1}. Knowitall database ID match **Calcite 84.87%**. Single, thin, rough fibre. Probably natural (cellulose) fibre.

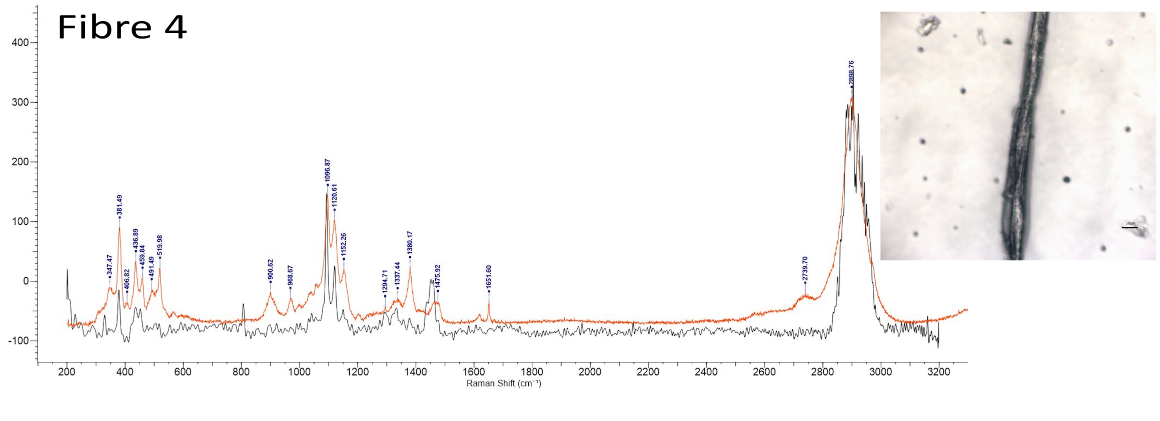

Figure S5: **Fibre 4 Raman spectrum:** Raman spectrum (black), and database PP spectrum (red). Raman spectrum: peaks at 380.12, 808.15, 1094.43, 1122.13, 1453.42 cm^{-1}. KnowItAll database: Noisy spectrum, peaks show some match to **cellulose 74.30%**. Irregular, rough fibre. Probably cellulose.

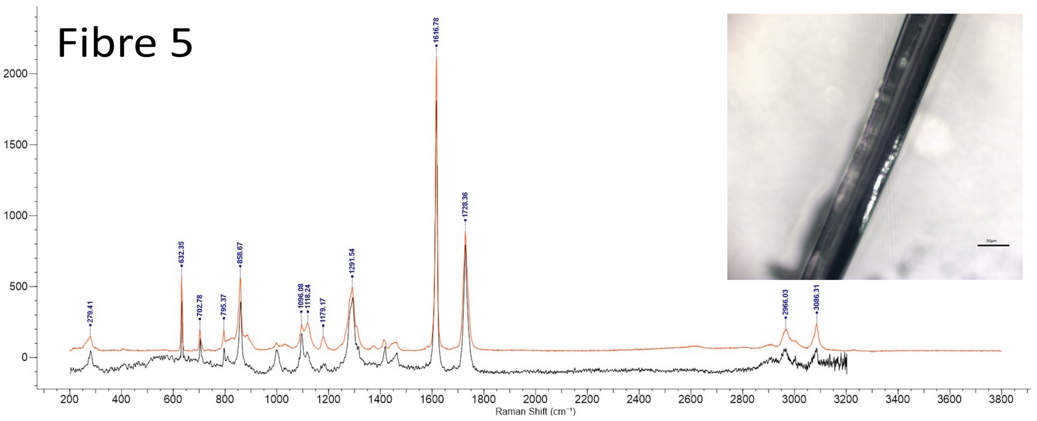

Figure S6: **Fibre 5 Raman spectrum:** Raman spectrum (black), and database PP spectrum (red). Raman spectrum: peaks at 279.41, 631.66, 702.78, 858.00, 997.81, 1093.84, 1289.63, 1417.21, 1614.45,1726.28 cm^{-1}. Knowitall database ID match **Polyethylene terephthalate (PET) 85.03%**. Single, thick, smooth fibre. Probably PET.

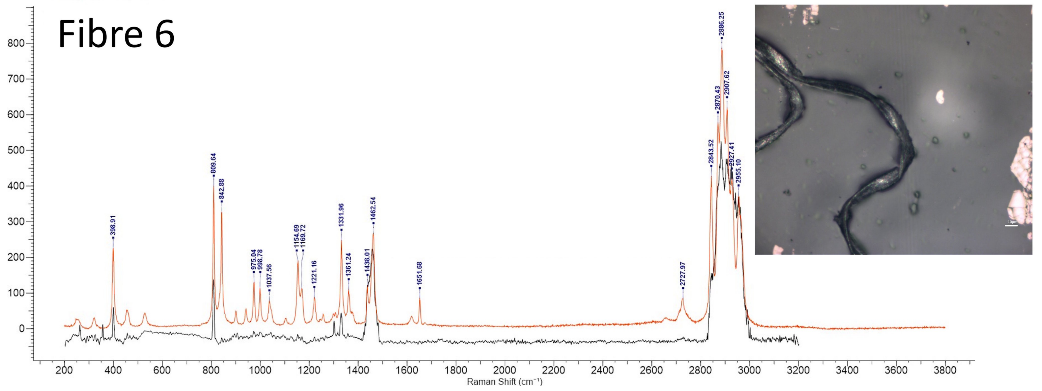

Figure S7: **Fibre 6 Raman spectrum:** Raman spectrum (black), and database PP spectrum (red). Raman spectrum: High background fluorescence and noisy spectra. Peaks at 399.45, 808.77, 840.64, 972.53, 998.11, 1044.05, 1152.26,1167.41, 1220.55, 1328.86,1358.02, 1457.51 cm^{-1}. KnowItAll database ID match: **polypropylene (PP) 77.03%**. Thin fibre cluster, probably PP.

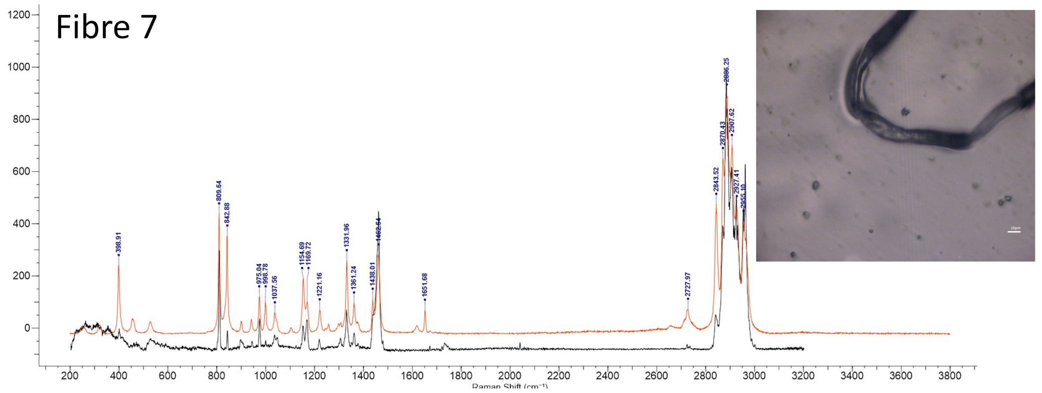

Figure S8: **Fibre 7 Raman spectrum:** Raman spectrum (black), and database PP spectrum (red). Raman spectrum: noisy spectrum, high background fluorescence. Peaks at 399.11, 809.08, 841.57, 896.33, 943.44, 973.14, 1035.44, 1150.21, 1167.12, 1217.68, 1329.14, 1359.96, 1459.97 cm^{-1}. Knowitall database ID match: **polypropylene (PP)** 73.97%.Thin, irregular fibre, probably PP.

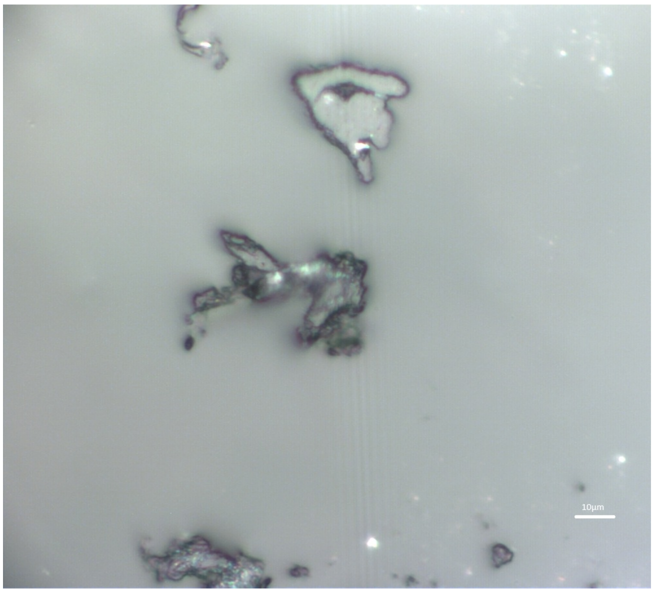

Figure S9: **Particle 8 microscopy:** Numerous irregular, shiny particles of 20 µm to 40 µm diameter. Very poor spectra only, no significant difference from background substrate. No match in Knowitall ID database. **No ID**. Probably sand grains.

S4 Fourier Transform Infrared Spectroscopy (FTIR)

Table S4: FTIR analysis of material extracted from Marimo. (HQI: Hit Quality Index)

Marimo sample	Particle number	Particle description	HQI	Library hit
M1-V	1	white fragment	0.58959	F17992.SP F17992 disperse blue 35 STD Fluka
	2	red fragment	0.47122	F87270.SP F872702,2,3,3-Tetrafluoro-1 Propanol Purum C3H4F4O
	3	black fibre	0.74219	Flour Standard
	4	blue dot	0.53578	Bromophenol Red
	5	clear fibre	0.96259	E.I.Dupont /Dacron (Polyester) Wh Fil Yarn 18-23U NBS-A0021
M1-W	1	white fragment	0.80408	Zinc Bacitracin Potassium Bromide disc
	2	white chunky fibre	0.70806	PVC additive Phthalate and Calcium Carbonate
	3	white fragment	0.58945	Copper(II) Trifluoromethanesulfonate
	4	dark fragment	0.59288	PVC additive Phthalate and Calcium Carbonate
	5	round white	0.65606	F79535.SP F795355-Phospho-D-Ribose 1 Diphosphate Sodium Salt
M1-X	1	white fragment	0.45322	Di-tert-Butylphenol
	2	white fibre	0.72514	Cellulose
	3	beige fragment	0.62777	PVC additive Phthalate and Calcium Carbonate
	4	white stick	0.70894	Chipboard P40 10.7%N 2.9/380MG KBR 0-00-0
	5	clear fragment	0.45842	Santoflex 13 Melt / Potassium Bromide (KBr) 0-00-0

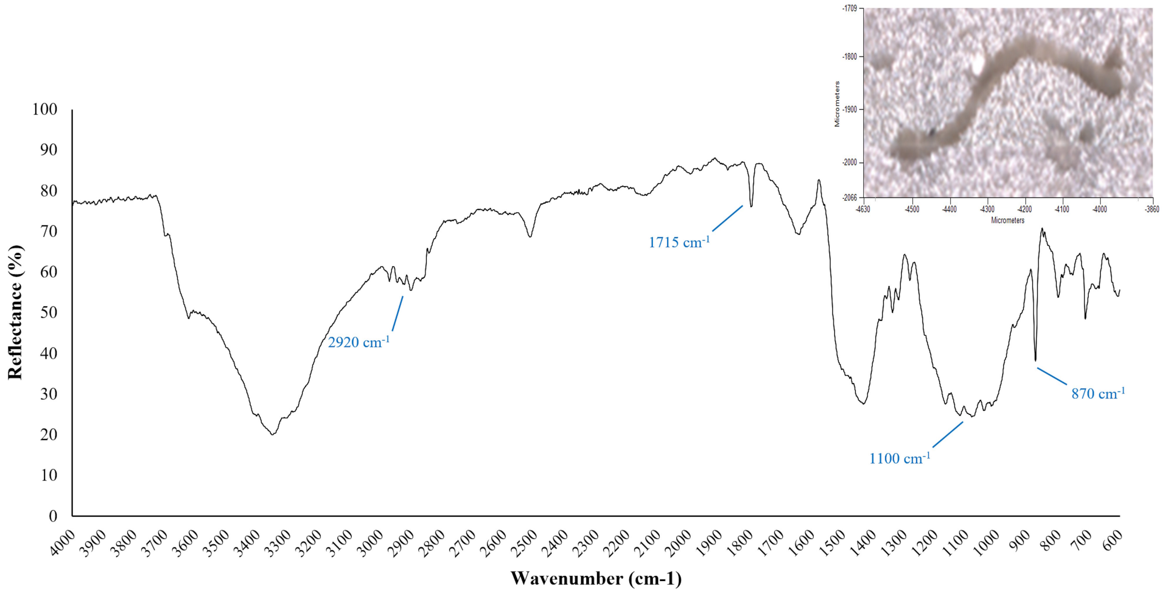

Figure S10: FTIR reflectance graph and image of white PVC fibre from Marimo sample M1-W. (HQI=0.71).

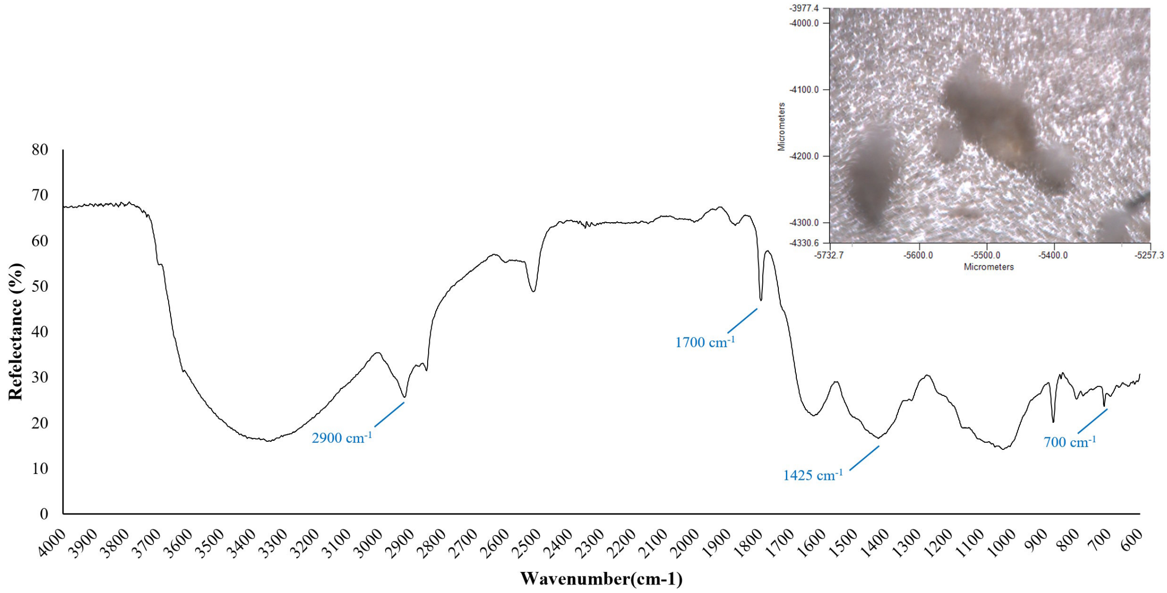

Figure S11: FTIR reflectance graph and image of a light brown PVC fragment from Marimo sample M1-X. (HQI=0.63).

S5 Marimo filtration

Table S5: Baseline bacterial loading enterococci in the environmentally derived surface water over one year (n=3 ± SD).

**Month**	**Enterococci (CFU 100 mL^{-1})**
January	0 ± 0
February	12 ± 7
March	3 ± 10
April	22 ± 15
May	238 ± 52
June	189 ± 59
July	185 ± 64
August	749 ± 298
September	1223 ± 1098
October	479 ± 92
November	70 ± 55
December	10 ± 8
